# Ni isotopes provide a glimpse of Earth’s pre-late-veneer mantle

**DOI:** 10.1126/sciadv.adj2170

**Published:** 2023-12-15

**Authors:** Yong Xu, Kristoffer Szilas, Lingyu Zhang, Jian-Ming Zhu, Guangliang Wu, Jie Zhang, Bin Qin, Yao Sun, D. Graham Pearson, Jingao Liu

**Affiliations:** ^1^ State Key Laboratory of Geological Processes and Mineral Resources, China University of Geosciences, Beijing, China.; ^2^ Department of Geosciences and Natural Resource Management, University of Copenhagen, Denmark.; ^3^ Department of Earth and Atmospheric Sciences, University of Alberta, Edmonton, Alberta, Canada.

## Abstract

Moderately siderophile (e.g., Ni) and highly siderophile elements (HSEs) in the bulk silicate Earth (BSE) are believed to be partly or near-completely delivered by late accretion after the depletion caused by metallic core formation. However, the extent and rate of remixing of late-accreted materials that equilibrated with Earth’s pre-late-veneer mantle have long been debated. Observing evidence of this siderophile element-depleted pre-late-veneer mantle would provide powerful confirmation of this model of early mantle evolution. We find that the mantle source of the ~3.8-billion-year-old (Ga) Narssaq ultramafic cumulates from Southwest Greenland exhibits a subtle ^60^Ni/^58^Ni excess of ~0.05 per mil and contains a clear HSE deficiency of ~60% relative to the BSE. The intermediate Ni isotopic composition and HSE abundances of the ~3.8-Ga Narssaq mantle mark a transitional Eoarchean snapshot as the poorly mixed 3.8-Ga mantle containing elements of pre-late-veneer mantle material transitions to modern Earth’s mantle.

## Teaser

3.8 Ga ultramafic rocks mark a transitional snapshot from the proto- to modern Earth’s mantle in the late accretion history.

## INTRODUCTION

Earth’s prolonged accretion, including the Moon-forming giant impact (*1*) and late veneer (*2*, *3*) events, all provided key inputs that evolved into the modern mantle. Late accretion is a critical process, which, although long invoked (*1*, *3*), remains poorly constrained, especially with respect to the time scale of mixing its signature back with Earth’s pre-late-veneer mantle. Elements with varying degrees of lithophile to siderophile affinity have proven effective in tracing different stages of Earth’s accretion, especially when their isotopes are used as tracers. For instance, lithophile O, Ca, Ti, and Nd record the full accretion history of Earth, while moderately siderophile Cr, Ni, and Mo reflect an accretionary history that is more biased toward early impacts (*4*). Among the best tracers of the late veneer, corresponding to the last ~0.5% of Earth’s mass (*3*, *4*), are the highly siderophile elements [HSEs; here including Re and five platinum group elements (PGEs): Os, Ir, Ru, Pt, and Pd]. The long-lived radioactive isotope systems (i.e., ^190^Pt-^186^Os and ^187^Re-^187^Os) embedded within the HSEs have also been widely used in dating planetary mantle differentiation and late accretion (*5*), especially the evolution of early Earth, such as the timing of late veneer (*6*), and the record of Hadean mantle melting (*7*). Among the moderately siderophile elements, Ni is particularly unusual because it behaves in a relatively refractory manner and has no change in chemical valence (Ni^2+^) during the volatile processes and magmatic evolution that drives planetary differentiation. Despite this, only a few studies have focused on the Ni stable isotopes of Earth’s early mantle (*8*). Recently, several studies (*9*–*13*) have attempted to define the Ni isotopic composition of the bulk silicate Earth (BSE), reporting distinguishable differences between modern BSE and chondrites, Earth’s building blocks. These studies have fermented widespread disagreement over whether the main driver of the observed Ni isotope fractionation in the silicate Earth was terrestrial core formation or late accretion. This controversy has led to studies involving high-pressure experiments (*14*), first-principles calculations (*10*), and determination of the Ni isotopic variation of ureilites relative to chondrites (*15*) as a means of approaching the problem. A more direct approach is to investigate early-Earth mantle-derived samples that did not receive or were not effectively mixed with the full complement of late-accreted materials (*16*–*19*) compared with other mantle materials.

**Fig. 1.**
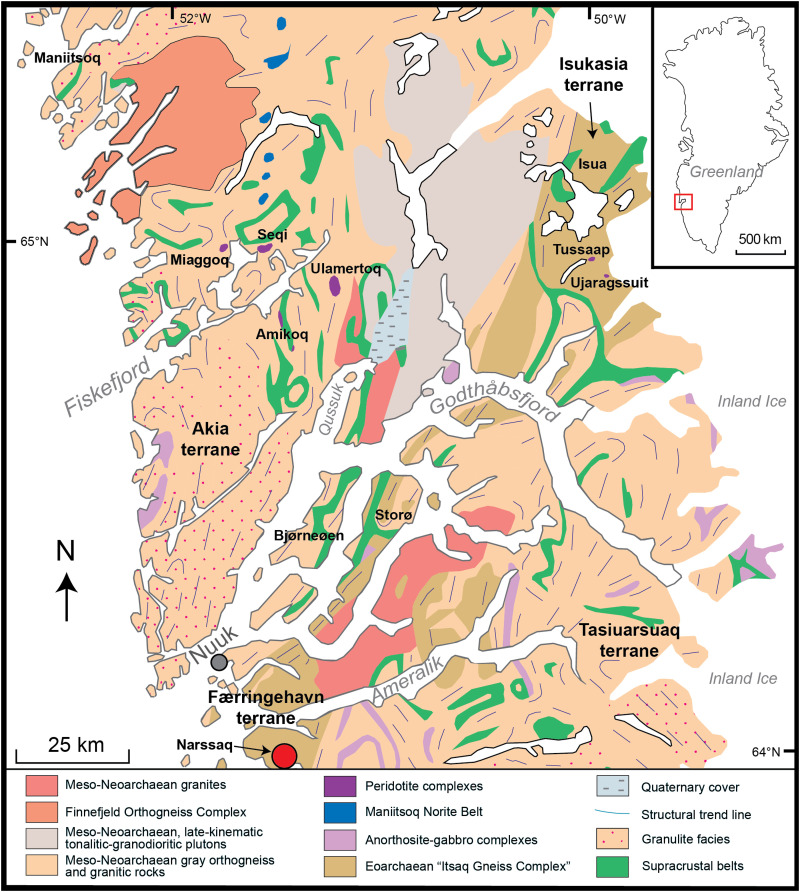
Simplified geological map of the IGC, Southwest Greenland. The main rock units exposed in the IGC are shown [modified after (*24*)]. The location of the Eoarchean NUB is marked by red solid circle.

Resolvable ^182^W/^184^W excesses (*16*, *18*) and suprachondritic Pt stable isotopic compositions (*17*) have hinted at a record of proto-Earth (pre-late-accretion) mantle domains contained within the Eoarchean rocks of the Itsaq Gneiss Complex (IGC), Southwest Greenland (Fig. 1). Specifically, Fischer-Gödde *et al.* (*19*) found that the ~3.8–billion-year-old (Ga) ultramafic enclaves in the IGC display a uniform excess in s-process Ru nuclides relative to the modern BSE, indicating that their mantle source was not yet fully equilibrated with late-accreted materials. Although the petrogenesis of the IGC ultramafic enclaves is still a matter of debate in terms of them representing mantle residues (*20*, *21*) or mafic/ultramafic melt cumulates (*22*–*24*), both origins involve material derived from Earth’s mantle in Eoarchean times, allowing us to constrain the Ni isotopic composition of the early terrestrial mantle. We should expect that rocks of either origin should retain their mantle signature because previous studies have shown that no quantifiable fractionation of Ni isotopes is generated by either mantle partial melting or fractional crystallization of mafic melts (*8*, *9*, *11*). Here, we present high-precision mass-dependent Ni isotopic data for 13 Eoarchean ultramafic rocks from the Narssaq ultramafic body (NUB) of the IGC, using these data to investigate the processes and potential time scale that led to the establishment of the subchondritic Ni isotopic signature of the modern BSE. Simultaneously, we report the PGEs and Re-Os isotopes for the same rocks, helping us to further identify the mixing history of late-accreted materials into the early terrestrial mantle.

## RESULTS

### Major and trace elements

We purposefully analyzed both fresh and altered peridotites to evaluate the potential role of secondary, low-T alteration on the Ni isotopic composition of ancient peridotites. Bulk-rock anhydrous major and trace element contents (table S1; Supplementary Materials) show that the three severely altered NUB peridotites containing abundant chlorites have high LOI (loss of ignition) values (3.2 to 8.6 wt %) and disturbed major and trace element systems. In contrast, the fresh NUB peridotites have relatively low LOI values (1.8 to 4.1 wt %; mean = 2.5 wt %), and major and minor element characteristics that vary due to igneous processes such as the strong correlations of Al, Fe, Ni, and Cr (*R*^2^ > 0.77) with MgO (34.2 to 49.2 wt %; fig. S1). Note that the NUB peridotites generally have higher FeO (8.3 to 15.4 wt %) than estimates of the primitive mantle (PM = 8.05 wt %; fig. S1), resulting in generally lower bulk Mg# [atomic Mg^2+^/(Mg^2+^+Fe^2+^) × 100, 79.9 to 91.3, average of 87.1] relative to depleted cratonic (91.9 ± 1.4) and sub-arc (90.5 ± 0.7) mantle (*25*). In addition, Ni [1165 to 2875 parts per million (ppm)] and Cr (2542 to 8508 ppm) concentrations of the NUB peridotites vary widely unlike typical mantle residues (fig. S1). Almost all the NUB peridotites except sample 208191 show nearly flat PM-normalized REE (rare earth element) patterns and remarkable negative Eu anomalies (fig. S2), which are very similar to, and overlap, the Isua dunites (*22*). Furthermore, their REE abundances are more than one order of magnitude higher than those of sub-arc mantle melting residues [e.g., the New Caledonia ophiolitic peridotites (*26*, *27*)].

### HSEs and Re-Os isotopes

The HSE abundances and Re-Os isotopic compositions are presented in table S1. All the NUB peridotites have highly fractionated chondrite (*28*)–normalized HSE patterns (fig. S2), consistent with Eoarchean ultramafic rocks that have been recently recognized as cumulates (*22*–*24*) but distinct from subduction-related mantle melting residues [e.g., sub-arc mantle xenoliths and SSZ (supra-subduction-zone) ophiolitic peridotites; compiled by (*22*)]. Moreover, their IPGE (iridium-group PGE: Os, Ir, and Ru) correlate positively, while PPGE (palladium-group PGE: Pt and Pd) correlate negatively, with indices of olivine accumulation (e.g., Ni; fig. S3). In addition, their chondrite-normalized Ru/Ir (Ru_N_/Ir_N_ = 1.27 to 13.9, mean = 7.44) ratios are mostly above 1.40, the primitive upper mantle (PUM) ratio (*29*), while their Pt_N_/Pd_N_ ratios (0.09 to 0.71, mean = 0.46) are all lower than the PUM value of 0.72 (fig. S4), corresponding to remarkable Ru and Pd positive anomalies (fig. S2). As important indicators of PPGE/IPGE fractionation, Pt_N_/Os_N_ (0.03 to 9.65, mean = 2.71) and Ir_N_/(Pt_N_ + Pd_N_; 0.04 to 4.36, mean = 1.06) ratios vary over three orders of magnitude (fig. S4). The NUB peridotites have very unradiogenic present-day ^187^Os/^188^Os ratios (0.1009 to 0.1075) relative to PUM estimates (0.1296) (*30*), corresponding to Re depletion model ages (*T*_RD_) between 3.0 and 3.9 Ga, with a mode at ~3.8 Ga (fig. S5). Their mantle model ages (*T*_MA_) vary to in excess of the age of Earth, with some samples yielding unrealistically low (<0.095) initial (at 3.8 Ga) ^187^Os/^188^Os ratios due to secondary Re addition, but most cluster around values expected for Eoarchean mantle-derived materials (0.100 to 0.102; table S1).

### Nickel stable isotopic compositions

**Fig. 2.**
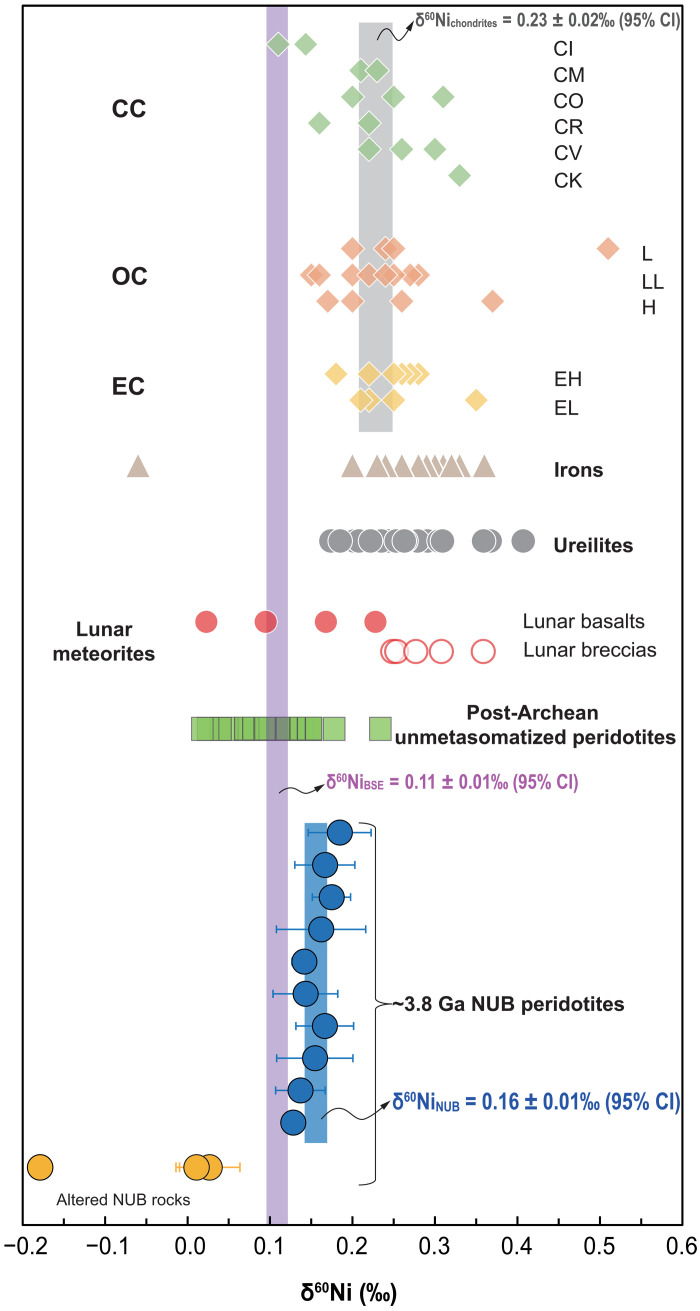
Mass-dependent Ni isotopic compositions of ~3.8 Ga Narssaq ultramafic rocks. The uncertainty bar represents 2SD. Blue, purple, and gray shaded fields represent the 95% CIs for the NUB sample group (δ^60^Ni = 0.16 ± 0.01‰; altered rocks are excluded from the mean), BSE estimate [δ^60^Ni = 0.11 ± 0.01‰; recalculated from 40 unmetasomatized post-Archean mantle peridotites; data from (*10*–*12*)] and different groups of chondrites (δ^60^Ni = 0.23 ± 0.02‰), respectively. The δ^60^Ni values of ordinary (OC), carbonaceous (CC), and enstatite (EC) chondrites; ureilites; iron meteorites; and lunar meteorites (*10*, *15*, *31*, *32*) are also shown for comparison.

The Ni isotopic compositions (expressed as δ^60^Ni, the per mil deviation of ^60^Ni/^58^Ni ratios relative to NIST SRM 986) of the NUB peridotites are listed in table S2. The three altered peridotites show very varied and much lighter δ^60^Ni values [−0.18 to 0.03 per mil (‰)], whereas the remaining 10 fresh rocks exhibit more limited δ^60^Ni variations ranging from 0.13 to 0.18‰, with an average of 0.16 ± 0.01‰ [95% confidence interval (CI); see Materials and Methods for details; Fig. 2]. The δ^60^Ni of the ~3.8-Ga NUB peridotites are clearly slightly heavier than that (δ^60^Ni = 0.11 ± 0.01‰, 95% CI) of the BSE value estimated from unmetasomatized post-Archean peridotites (Fig. 2) (*10*–*12*) but obviously lighter than the average (δ^60^Ni = 0.23 ± 0.02‰, 95% CI) of bulk chondrites (*10*, *15*, *31*, *32*) (Fig. 2). These observed differences calculated through both the Welch’s *t* test (*P* << 0.05) and Mann-Whitney *U* test (*P* << 0.05) are statistically significant.

**Fig. 3.**
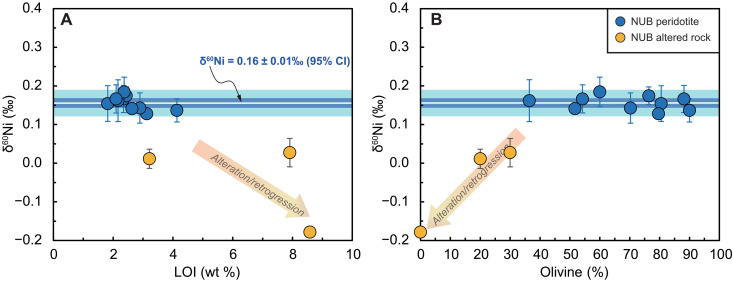
Relationship between Ni stable isotopic compositions and indices of alteration/retrogression and olivine accumulation. Bulk-rock δ^60^Ni values versus LOI (**A**), and modal olivine abundance (**B**) for ~3.8-Ga NUB peridotites and altered rocks. Light and dark blue shaded fields represent the 2SD (±0.03‰) and 95% CI (±0.01‰) for the NUB sample group, respectively.

**Fig. 4.**
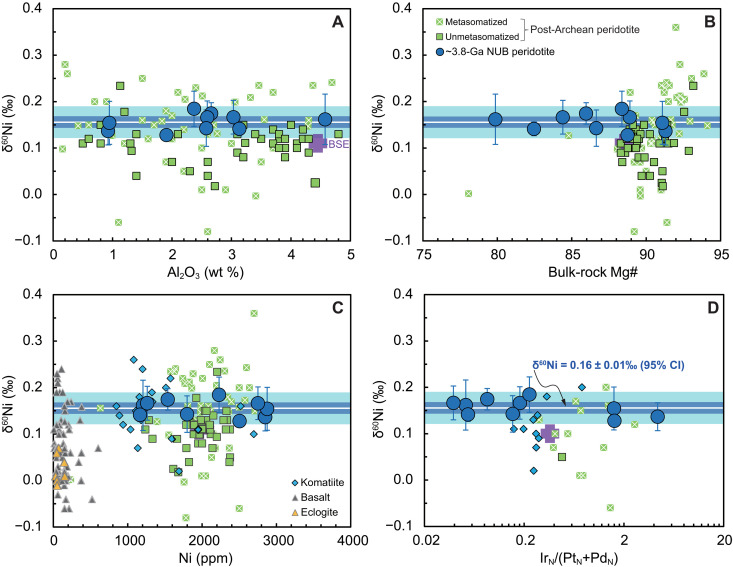
Limited variations of δ^60^Ni values relative to change of lithophile or siderophile indices of silicate and sulfide partial melting, fractional crystallization. Bulk rock δ^60^Ni values versus Al_2_O_3_ content (**A**), bulk rock Mg# (**B**), Ni concentration (**C**), and Ir_N_/(Pt_N_ + Pd_N_) (**D**) for ~3.8-Ga NUB peridotites. BSE estimate (*29*, *76*), and data of post-Archean peridotites, komatiites, eclogites, and basalts from literature (*9*–*13*, *44*) are also plotted for comparison. Light and dark blue shaded fields represent the 2SD (±0.03‰) and 95% CI (±0.01‰) for the NUB sample group, respectively.

The δ^60^Ni of the fresh NUB peridotites are invariant with respect to LOI values or modal mineral (olivine, amphibole, and chromite) abundances (Fig. 3 and fig. S6), while the δ^60^Ni of the highly altered NUB peridotites decrease with increasing LOI and modal chlorite abundance and with decreasing olivine modal abundance. Important indices of silicate partial melting and fractional crystallization such as lithophile Al, Mg#, and Ni concentrations show no systematic correlations with δ^60^Ni for the fresh NUB peridotites (*R*^2^ ≤ 0.15; Fig. 4). In addition, absolute HSE abundances (fig. S7) and chondrite-normalized ratios [e.g., Ir_N_/(Pt_N_ + Pd_N_) and Pt_N_/Os_N_] that reflect fractionation among HSEs, together with *T*_RD_ ages (Fig. 4 and fig. S4) exhibit no correlation with the limited δ^60^Ni variations (*R*^2^ ≤ 0.29).

## DISCUSSION

### Petrogenesis of the Narssaq ultramafic rocks: Mantle residues or cumulates?

The good correlations and large variability of key major and minor element contents with MgO (fig. S1), as well as the covariations between PGE and Ni (fig. S3), indicate that the bulk compositions of the fresh NUB peridotites are mainly controlled by igneous processes rather than secondary alteration. However, it is difficult to conclusively identify whether these correlations reflect an origin as melt residues of mantle partial melting processes or whether they arise from cumulate processes acting on ultramafic-mafic magmas. Similar to the ~3.8-Ga Isua dunites (*22*), the Eoarchean NUB peridotites also have nearly flat PM-normalized REE patterns (fig. S2), indicating that they are not simple analogs of Archean mantle residues [see the review in (*33*)]. Although previous studies demonstrated that this kind of light- and moderate-REE enrichment can be explained by the infiltration of subduction-related fluids/melts into initially depleted peridotites (*21*), there are other features that cannot be reconciled with an origin of sub-arc mantle in a subduction environment. For example, all the NUB peridotites display abnormally elevated FeO contents (up to 15.4 wt %) relative to the PM, far above even fertile mantle estimates and highly atypical for mantle residues from any tectonic setting (fig. S1). Moreover, compared to mantle peridotites whose compositions are dominated by partial melting ± metasomatism, the Cr and Ni concentrations of the NUB peridotites vary over considerably wider ranges, well outside the range of typical melting residues (fig. S1). In addition to metasomatism from various melts/fluids, the sub-arc mantle may also experience a high-degree partial melting due to the assistance of water, thus leading to strong fractionation in heavy REE (HREE), as illustrated by the New Caledonia ophiolitic peridotites (*26*, *27*). However, the HREE for the NUB peridotites range from slightly enriched to variously depleted relative to the PM, but with little to no inter-element fractionation (fig. S2), which cannot be simply explained by melt metasomatism because of the incompatibility of HREE during shallow melting and their compatibility during deep melting. As for the highly fractionated chondrite-normalized HSE patterns (fig. S2), especially for the sharp anomalies of Ru and Pd, the NUB peridotites are in notable contrast to the ranges of mantle-wedge peridotites. Mantle peridotites formed in subduction zones typically have relatively lower Os_N_/Ir_N_ and more radiogenic ^187^Os/^188^Os ratios owing to high-*f*O_2_ metasomatism and slab-derived contamination (*34*). The HSE systematics of the NUB peridotites obviously do not fall into this category (fig. S4). Similar to the Archean Pilbara komatiites (*35*) and Isua cumulates (*22*), the PGE abundances of the NUB peridotites are highly correlated with indices of olivine fractional crystallization (e.g., MgO and Ni; fig. S3). These positively correlated trends of IPGE and negatively correlated trends of PPGEs against MgO or Ni, also referred to as olivine control lines, are believed to be the result of sulfide-undersaturated magma differentiation dominated by the combined control of olivines and PGE alloys (*35*, *36*).

The geochemical characteristics described above, however, are not unique to the Eoarchean NUB peridotites but shared by many ultramafic rocks from the Eoarchean terranes in Southwest Greenland [e.g., the Isua supracrustal belt (ISB) in Isuakasia Terrane (*22*, *24*)] and West Australia [East Pilbara Terrane (*23*)]. Recent studies have realized that these Eoarchean ultramafic rocks do not represent sub-arc mantle residues but are rather igneous cumulates, which formed by the accumulation of olivine + chromite (accompanied with PGE alloys), plus subsequent interaction with the co-genetic liquids (*22*–*24*). In addition to abnormally elevated FeO and highly varied Cr and Ni, their cumulate petrogenesis is further supported by MELTS modeling of the liquid evolution and the corresponding bulk cumulates during fractional crystallization of olivine and spinel (*24*). Accordingly, we interpret the NUB peridotites as being crystallized from (ultra)mafic magmas extracted from large-degree mantle melts.

### Extent and timing of late veneer in the NUB mantle source

**Fig. 5.**
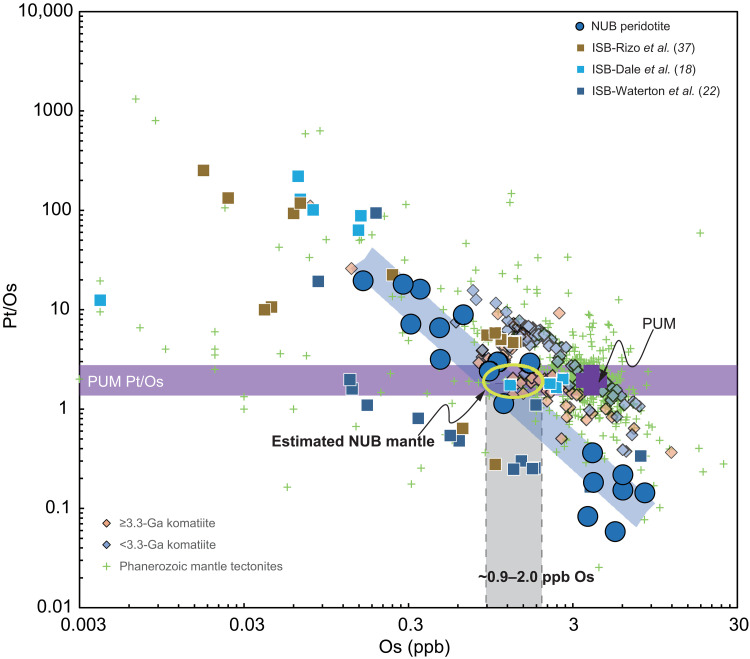
Ratio of moderately compatible HSE (Pt) to highly compatible HSE (Os), plotted against the denominator. Data of ≥3.3-Ga and <3.3-Ga komatiites (*38*, *77*–*79*), Phanerozoic mantle tectonites (*80*), and ISB ultramafic rocks (*18*, *22*, *37*) are also shown for illustration.

Recent HSE data for the Greenlandic Narssaq, Isua, and south of the ISB ultramafic rocks have been interpreted to reflect that the NUB mantle source is dominated by between at least 60 and>95% of late-accreted material with a chondritic composition, which was efficiently admixed (*6*, *37*). However, given that both the NUB and Isua dunites are more likely to be ultramafic cumulates rather than pure mantle fragments, their HSE data cannot be directly used to determine the HSE abundances of their mantle sources. Dale *et al.* (*18*) introduced an effective way of quantifying the effect on mantle sources using ratios between two HSEs of differing compatibility, plotted against the denominator [e.g., Pt/Os versus Os; Fig. 5]. This approach defines arrays for mantle melts that emanate from loci that closely match the HSE characteristics of the original source. This method is applicable to both mantle residues and ultramafic cumulates when taking into account differences in partitioning, source mineralogy, and crystallizing mineral assemblage because such differences should result in variable gradients of the arrays rather than differences in apparent source composition.

Here, we apply our compiled HSE data [*n* = 18, from this study and those from (*6*)] to reevaluate the extent of the late veneer in the NUB mantle source. Taking Pt/Os versus Os as an example (Fig. 5), we find that post–3.3-Ga komatiites and Phanerozoic mantle tectonites commonly define an array that broadly emanates from the PUM Os concentration [Os_PUM_ = 3.9 ± 0.5 parts per billion (ppb) (*29*)], suggesting a mantle source equivalent to the BSE in terms of HSE abundances. In contrast, both the NUB peridotites and ISB ultramafic rocks (*18*, *22*, *37*) plot outside this array, instead lying closer to pre–3.3-Ga komatiites, for which HSE-poor mantle sources were previously suggested (*35*, *38*–*41*). Furthermore, the intersection of the array defined by the NUB peridotites and the Pt/Os ratio of PUM suggests that these ultramafic cumulates were derived from an Os-poor mantle source with ~0.9 to 2.0 ppb Os, equivalent to 23 to 51% of the modern BSE estimate (*29*). Using this method, we further estimate that the NUB mantle source may have ~0.6 to 1.6 ppb (17 to 46% of PUM) Ir and ~2.0 to 4.0 ppb (26 to 53% of PUM) Pt (fig. S8), both of which are obviously lower than those of the BSE budget (Ir_PUM_ = 3.5 ± 0.4 ppb; Pt_PUM_ = 7.6 ± 1.3 ppb). On the basis of estimated Os, Ir, and Pt concentrations, the NUB mantle source contains only ~36 ± 8% of the full late veneer HSE component. This estimate of the proportion of material added during late accretion is slightly lower than the previous estimate of ~58 ± 6% (*18*). Our estimate of the HSE-poor NUB mantle source is consistent with the evidence from well-resolved excesses in mass-independent ɛ^100^Ru (0.22 ± 0.04, 95% CI) found in these peridotites (*19*), both of which unequivocally suggest that the mantle source of the Narssaq ultramafic cumulates did not receive the full complement of late veneer materials.

The Re-Os radioactive isotope system is robust in dating mantle-derived magmatic events in early Earth, which can help us estimate the timing of late veneer addition to the NUB mantle source. Owing to Re mobilization during secondary alteration since their formation, neither a Re-Os isochron nor an Al-Os pseudoisochron is obtained for the NUB peridotites. Rhenium depletion Os model ages (*T*_RD_) and mantle Re-Os model ages (*T*_MA_) become the sole choice. With the exception of several unrealistic (>Earth’s age) ages, and two *T*_RD_ ages around 3.0 Ga that are likely due to the disturbance by the late Archean (~2.7 Ga) amphibolite to granulite facies metamorphism (*42*), many of the Re-Os model ages are clustered within the Eoarchean. *T*_MA_ ages for most NUB peridotites are older than the corresponding *T*_RD_ ages in the same rocks, indicating that Re addition and/or that Re was not completely consumed. In a kernel density estimation (KDE) plot of *T*_RD_ ages (fig. S5), the prominent mode at ~3.8 Ga likely represents a minimum estimate of the formation time because this signature will be a mixture of depleted mantle and newly accreted material. Furthermore, the minimum estimate of ~3.8 Ga is consistent not only with the U-Pb ages (3.65 Ga) of cross-cutting tonalitic-trondhjemitic-granodioritic (TTG) intrusives (*43*) but also with the whole-rock Lu-Hf isochron ages (3.93 ± 0.15 Ga) obtained from the NUB peridotite enclaves (*21*). Therefore, all the age data suggest that the NUB peridotites were formed at least 3.8 billion years ago, when the onset of incomplete (~36 ± 8%) late accretion was initiated.

### Nickel isotopic composition of the NUB mantle source

Previous studies have revealed that weathering and secondary hydrous alteration processes acting on silicate rocks can cause large Ni isotopic fractionation, usually leaving altered ultramafic rocks isotopically light (*8*). Hence, the three strongly altered NUB rocks displaying remarkably light Ni isotopic compositions (δ^60^Ni from −0.18‰ to 0.03‰; Fig. 2) are excluded from the following discussion, which focusses on the “primary” signal. The petrography (fig. S9) of the remaining “fresh” NUB peridotites contains little of the serpentinite and magnetite mineral association typical of many ultramafic rocks from Phanerozoic ophiolites. In these unaltered NUB peridotites, δ^60^Ni values do not vary with LOI (Fig. 3), suggesting that their Ni isotopic compositions were not disturbed by weathering/low-T alteration processes. Given their δ^60^Ni values, any unidentified alteration would mean that these remaining values are minima, although the homogeneity of their compositions supports the contention that they represent magmatic δ^60^Ni values.

The behavior of Ni isotopes during metamorphism has not been well studied; however, basic magmatic rocks subjected to eclogite facies metamorphism have similar δ^60^Ni (0.02 ± 0.06‰) to unmetamorphosed basalts (0.03 ± 0.16‰) (*10*), suggesting that the influence of metamorphism on Ni isotopic composition is relatively limited. Moreover, there is no association between the limited presence of metamorphic amphibole and minerals indicative of minor retrogression/alteration (e.g., chlorite; fig. S9) and variations in δ^60^Ni in the NUB peridotites (fig. S6), further demonstrating that their Ni isotope characteristics have not been effectively fractionated by metamorphic processes.

The highly uniform Ni isotopic compositions displayed by the freshest NUB peridotites (δ^60^Ni = 0.16 ± 0.01‰, 95% CI) cannot be linked to variability in any indices of magmatic evolution (e.g., Al_2_O_3_, Mg#, and Ni; Fig. 4), indicating that no evident Ni isotopic fractionation occurred during either partial melting of the mantle source or the fractional crystallization process that created these peridotites. Previous studies (*9*–*12*) have suggested that silicate partial melting or melt depletion cannot noticeably fractionate isotopes of Ni, which is present only as Ni^2+^ in rock-forming minerals (e.g., olivine, pyroxene, and spinel). Many lines of evidence support this claim, for example, (i) there is no covariation between the Ni isotopic composition and parameters such as modal clinopyroxene content, bulk-rock Al_2_O_3_ concentration, Nb/Zr ratio, and Na_8.0_ value (the Na_2_O content at MgO = 8.0 wt %) in either global mantle peridotites or basalts (*9*, *11*, *12*). (ii) Modeling suggests a maximum Ni isotope fractionation of 0.15‰, for which the residual peridotite δ^60^Ni would be buffered at a constant composition (*11*).

Studies on the behavior of Ni stable isotopes during the process of silicate fractional crystallization or accumulation are relatively limited. Saunders *et al.* (*9*) found that throughout the well-defined trend of olivine crystallization defined by MgO and Ni in basalts from different settings, there is no accompanying variation in δ^60^Ni. For the NUB peridotites, we also observe no variation in bulk δ^60^Ni relative to modal olivine (Fig. 3) and chromite (Cr-spinel) contents (fig. S6), suggesting an insignificant net effect of olivine + chromite accumulation on Ni isotopic fractionation, regardless of the fact that spinel is isotopically heavier than olivine (*12*, *44*).

Despite the minimal influence of silicate partial melting or fractional crystallization on Ni isotopes, unusual and relatively large Ni isotopic fractionations up to 1.2‰ have been observed between silicates and sulfides in ultramafic-mafic magmas associated with magmatic sulfide ore deposits (*45*–*48*). A recent theoretical model of fractionation between komatiitic lava and sulfide melt with Ni isotopic exchange followed by sulfide crystallization predicts a range of δ^60^Ni values from +0.17‰ to −1.02‰ in sulfide-rich rocks depending on the extent of fractional crystallization and the amount of trapped melt between sulfide minerals (*47*). More recently, a rather large Ni isotopic fractionation with δ^60^Ni varying from +0.07 to +1.00‰ was recognized in Noril’sk basalts and can be well explained by sulfide-silicate liquid immiscibility during magmatic differentiation where light isotopes preferentially partition into the sulfide liquid (*48*). In addition, sulfide metasomatism and dissolution have also been proposed to explain lighter Ni isotopic signatures in some peridotite xenoliths (*11*) and nephelinitic melts (*44*), respectively. Accordingly, there is no doubt that sulfide is a key phase affecting Ni isotopic compositions in ultramafic-mafic rocks, so its influence must be evaluated. However, the negligible modal sulfide (fig. S9) present in the NUB peridotites could not be a major phase in hosting the bulk Ni budget (1165 to 2875 ppm). The bulk-rock Ni budget is largely dominated by olivine with high modal proportions (with an average of 71%) and high NiO contents (0.2 to 0.4 wt %; table S3), which is also illustrated by the positive correlation of bulk-rock Ni concentrations with modal proportions (*R*^2^ = 0.78) or average Ni contents (*R*^2^ = 0.94) of olivine (fig. S10). By using the measured average compositions (table S3) and modal abundances (table S1) of major minerals, olivine is quantitatively assessed as the dominating phase for the bulk Ni budget, with the proportions accounting for the bulk-rock Ni reservoir ranging from 70 to 100% (fig. S11), followed by amphibole (<13%) and chromite (<10%). This assessment also suggests that the proportions of sulfides accounting for the bulk Ni budget must be very limited.

The absolute and relative abundances of PGEs that are highly siderophile and strongly chalcophile (*49*) can help us further evaluate the role of sulfide during the evolution of the NUB peridotites and hence the influence on Ni isotopic compositions. The PPGE abundances show a general negative trend against bulk Ni, while the IPGE show a positive trend (fig. S3), indicating that olivine was the only major fractionating phase linked with PGE alloys, instead of sulfides, in controlling the behavior of PGEs during sulfide-undersaturated magma differentiation (*35*). Even if both the remarkably fractionated PGE patterns (fig. S2) and varied relative PGE abundances [e.g., Os_N_/Ir_N_, Ru_N_/Ir_N_, Pt_N_/Pd_N_, Pt_N_/Os_N_, and Ir_N_/(Pt_N_ + Pd_N_) ratios; Fig. 4 and fig. S4] reflect the involvement of sulfides and/or alloys, these PGE variations and absolute PGE abundances (fig. S7) are not associated with limited change of δ^60^Ni values, still suggesting that no process such as incongruent melting, immiscibility, crystallization, or metasomatism of sulfides (*44*, *47*, *48*, *50*) has effectively affected the Ni isotopic compositions of the NUB peridotites. Although samples 208191 and 208196 have a few visible pentlandites (fig. S9), they also show δ^60^Ni values and PGE patterns identical to other NUB samples (figs. S2 and S4), further supporting the insignificant role of sulfide fractionation in controlling the Ni isotopic composition of the NUB peridotites.

In summary, we find no evidence that the Ni isotopic systems of the ~3.8-Ga NUB peridotites were affected by high-temperature magmatic processes and secondary alteration. We thus infer that their Ni isotopic compositions reflect their mantle source region, which, at 3.8 Ga, is characterized by δ^60^Ni = 0.16 ± 0.01‰, in a mantle source region that contains a partial (~36 ± 8%) inventory of late-accreted HSEs and an s-process Ru excess relative to the BSE (*19*).

### Establishment of a subchondritic Ni isotopic signature in Earth’s mantle

Whether the Ni isotopic composition of the BSE is chondritic or not is of great importance for characterizing Earth’s building blocks, especially for the source of late-accreted materials that supplemented the Ni deficit sequestered by core formation. In the first systematic study of Ni isotopes on mantle xenoliths rather than just reference materials, Gall *et al.* (*13*) constrained the mass-dependent Ni isotopic composition of the BSE to be chondritic (δ^60^Ni = 0.23 ± 0.06‰), suggesting little to no Ni isotopic fractionation was produced in Earth’s mantle from core formation. However, subsequent studies have argued against this chondritic Ni isotopic composition because some severely metasomatized peridotites that do not represent the BSE were included in the analyzed suite. Acquiring more data, Klaver *et al.* (*11*) subsequently observed a difference between their estimate of the isotopically light BSE (δ^60^Ni = 0.11 ± 0.01‰, 95% CI) and chondrites (Δ^60^Ni_Chondrites-BSE_ ≈ 0.13 ‰), which they thought likely resulted from Earth’s core segregation. In contrast, Wang *et al.* (*10*) demonstrated, on the basis of first-principles calculations and previous experimental studies (*14*, *51*), that core formation cannot produce such a light Ni isotopic composition for the BSE because limited Ni isotopic fractionation during core formation at high temperatures and pressures is predicted from these approaches. Whereafter, it was discovered that the main group ureilites have similar δ^60^Ni (0.26 ± 0.03‰) as chondrites (0.23 ± 0.02‰) (*15*). If ureilites represent “processed” planetary materials that have experienced core formation, then this observation also supports the notion that core formation does not observably fractionate Ni stable isotopes.

In contrast to these predictions for minimal high-T/P fractionation of Ni isotopes during core formation in large planetary bodies (e.g., Earth), it was more recently reported that low-pressure and low-temperature core-mantle differentiation within achondrites (e.g., Vesta) from small differentiated bodies can potentially produce relatively large Ni isotopic fractionation (*32*), leaving their planetary mantle isotopically light. In a further extension of these results, it was proposed that an aubrite-like differentiated planetary body (Theia) whose mantle was enriched in light Ni isotopes, collided with, and merged into the proto-Earth during the Moon-forming giant impact (*10*, *32*), leading to a lowering of Earth’s mantle from an approximately chondritic composition (δ^60^Ni = 0.23 ± 0.02‰) to the modern estimate (δ^60^Ni = 0.11 ± 0.01‰) for the BSE. Some researchers (*31*) have cast doubt on whether the proto-Earth was chondritic in Ni isotopes, introducing other possible scenarios such as chondrule-rich accretion (*15*) and nebular fractionation (*52*), but these models are difficult to reconcile with isotopic evidence from lithophile to siderophile systems that support a chondritic character to Earth’s building blocks (*1*, *3*, *4*). Although still controversial, on the basis of the similarities of nucleosynthetic (mass-independent) isotopic compositions of both lithophile and siderophile elements (e.g., O, Ti, Nd, Cr, Ni, Mo, and Ru), enstatite chondrite or enstatite chondrite-like differentiated planetary bodies were considered as the most likely building blocks for Earth (*4*, *19*, *53*).

Our measurements of the Ni isotopic composition of the Eoarchean Greenlandic NUB peridotites can help resolve this debate because these rocks derive from a mantle source that is a part mixture of early mantle that experienced core formation and late veneer materials, as explained above. Hence, these ultramafic rocks represent an important connecting point between the proto-Earth mantle, before core formation, and the modern BSE composition. The NUB mantle has a Ni isotopic composition that is subchondritic (δ^60^Ni = 0.16 ± 0.01‰) and slightly heavier than the δ^60^Ni of modern BSE (δ^60^Ni = 0.11 ± 0.01‰; Fig. 2). This result is, in part, consistent with the previous predication that the early (after core formation) BSE was already displaying a subchondritic Ni isotopic signature before the late veneer (*10*, *32*). Nevertheless, it is unclear whether the subchondritic Ni isotopic signature of the present BSE was created solely by a singular large-impact event or gradually formed by continuous multistage accretion.

**Fig. 6.**
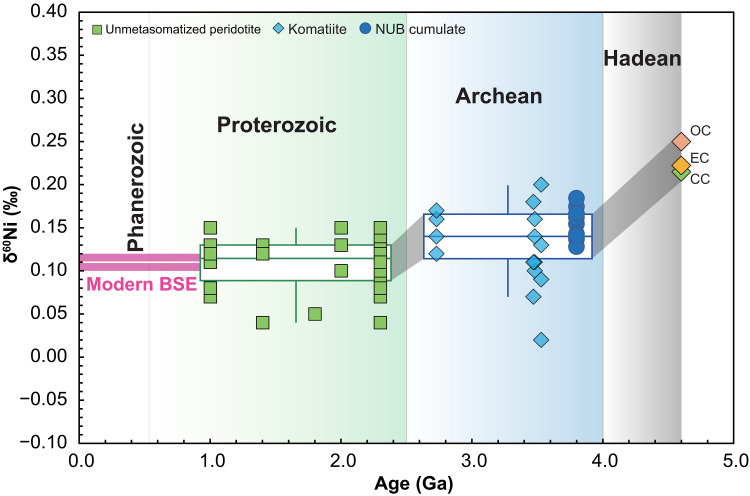
δ^60^Ni values versus ages of ultramafic rocks. Including ~3.8-Ga NUB rocks (*n* = 10) first reported in this study, and komatiites [Barberton-3.5 Ga (*n* = 11) and Ontario-2.7 Ga (*n* = 4)] and post-Archean unmetasomatized mantle peridotites [Kuandian-2.3 Ga (*n* = 6), Turon de Tecouere-2.3 Ga (*n* = 8), Vitim-2.0 Ga (*n* = 4), Damaping-1.8 Ga (*n* = 1), Zabargad-1.4 Ga (*n* = 3), Tariat-1.4 Ga (*n* = 2), and Horoman-1.0 Ga (*n* = 9)] from the literature. Data sources for the age and δ^60^Ni are listed in table S5 (Supplementary Materials). The box-and-whisker plots for the Archean and post-Archean ultramafic rocks and 95% CI for the modern BSE are also shown for comparison.

Despite these model uncertainties, our reported data, combined with previous data, enable us to outline the temporal change of the Ni isotopic composition of the BSE (Fig. 6). The resulting trend demonstrates that the subchondritic Ni isotopic composition of the terrestrial mantle was established by multiple processes rather than one sudden change. Our model starts with chondrites as Earth’s building blocks, based on isotopic similarities for both lithophile and siderophile elements (*1*, *3*, *4*). Hence, we assume that the Ni isotopic composition of the proto-Earth mantle is also chondritic (an average of ordinary chondrite, enstatite chondrite, and carbonaceous, with δ^60^Ni = 0.23 ± 0.02‰) because of the limited effect of core-mantle differentiation in a large planetary body such as the proto-Earth. From the beginning of Earth’s formation through the Hadean to early Eoarchean times, the Ni isotopic composition of the BSE changed markedly, from a chondritic composition, to δ^60^Ni = 0.16‰ based on the mantle value indicated by the NUB peridotite compositions (Fig. 2). This offset from chondrite (Δ^60^Ni_chondrites-NUB_ ≈ 0.07 ‰) is smaller than estimates from previous studies (*10*, *11*, *31*), suggesting Δ^60^Ni_chondrites-BSE_ ≈ 0.13 ‰, enabling us to relax the previous constraint on the Moon-forming giant impactor (*10*). On the basis of mass balance, if using the older estimates of pre–late accretion mantle δ^60^Ni, the materials accreted to the proto-Earth mantle would need to have δ^60^Ni values as low as −0.35‰. Instead, if using the NUB mantle estimate of δ^60^Ni = 0.16‰ for the 3.8 Ga pre-accretion mantle, the late-accreted material requires a δ^60^Ni value of ~−0.1‰, which, when mixed with the proto-Earth mantle, would be enough to account for the present-day BSE value (fig. S12). Notwithstanding mass balance arguments, we are inclined to accept that the Moon-forming impact was the primary cause of the shift in the Ni isotopic composition of terrestrial mantle from chondrite-like to subchondritic because this event may have contributed more than 20% to the Ni budget of the BSE (*54*). Such a model is in good agreement with the observation that lunar basalts also have systematically lighter Ni isotopes than chondrites (Fig. 2) (*32*). Assuming that the positive nucleosynthetic Ru isotopic anomaly (ɛ^100^Ru = 0.22 ± 0.04) (*19*) and light mass-dependent Ni isotopic composition (δ^60^Ni = 0.16 ± 0.01‰) of the NUB mantle source were both imparted by the Moon-forming impactor, this signature is not represented by any known chondritic meteorites because they all exhibit negative nucleosynthetic Ru isotopic anomalies (*55*) and heavy mass-dependent Ni isotopic compositions (Fig. 2) (*10*, *15*, *31*, *32*). Nevertheless, both the s-process Ru excess and isotopically light Ni exhibited by Earth’s pre-late-veneer mantle require that the building materials (i.e., moon-forming impactor) that contributed to the early stages of Earth’s growth came from the innermost region of the Solar System characterized by a reduced, noncarbonaceous component (*10*, *19*). Although the exact source for the light-δ^60^Ni impactor remains enigmatic, mass-independent Ni isotopic studies on chondrites and terrestrial samples (*56*–*58*) may shed light on this divergence. For example, carbonaceous and ordinary chondrites show clear, well-resolved positive and negative nucleosynthetic Ni isotopic anomalies, respectively, while enstatite chondrites have near-zero ɛ^62^Ni values that are identical to the BSE estimate, possibly indicating that the accreted materials accounting for >20% BSE’s Ni might originate from an enstatite-like source region in the inner protoplanetary disk. However, this notion is inconsistent with the large discrepancy between the BSE and enstatite chondrites in terms of mass-dependent Ni isotopes (Fig. 2), implying that more complex processes, beyond/in addition to late accretion were involved. We believe that mass-independent Ni isotopic analyses of more terrestrial geological rocks before and after the late accretion and even lunar rocks, followed by systematic comparisons, can more accurately define the source of the incorporated building materials.

From the temporal trend defined in Fig. 6, a second change in the Ni isotopic composition of Earth’s mantle occurred between the Archean and Proterozoic, with a δ^60^Ni from 0.16‰ subtly reduced to 0.11‰. We consider two possibilities for this δ^60^Ni decrease: The first is the accretion of a late veneer that contributed <5% of Ni budget of the BSE (*3*), but this option is not supported by the carbonaceous chondrite-like isotopic signature for the late veneer inferred from previous studies (*1*, *3*, *19*, *59*–*61*) and is also inconsistent with the observation that lunar breccias that were contaminated by the impact of chondritic debris have homogenously chondritic Ni isotopes (Fig. 2) (*32*). The second option is that pre-late-veneer accreted materials, with isotopically lighter Ni such as those measured in the NUB mantle, were heterogeneously distributed and took a long time to fully homogenize with the entire mantle. This type of progressive evolution of Earth’s mantle via sluggish in-mixing of extraterrestrial materials has previously been suggested on the basis of a broadly progressive increase in PGE concentrations with time indicated by the Archean komatiites from the Pilbara and Kaapvaal cratons (*38*). Our estimate of the presence of only ~36 ± 8% component of late-accreted HSEs (Fig. 5) in the NUB peridotites along with the observed s-process Ru excess (*19*) indicates that the ~3.8-Ga NUB mantle source did not receive a complete complement of chondritic accretion. This raises the clear possibility that the mantle beneath the Southwest Greenland lithosphere at 3.8 Ga preserved some isotopically heavier Ni remaining after core-mantle differentiation. This isotopically heavier Ni signal is also seen in Archean komatiites but rarely observed in the post-Archean unmetasomatized mantle peridotites (Fig. 6). This crucial transition could represent the beginning of large-scale mantle convection toward the end of the Archean, coincident, for example, with the onset of modern-style plate tectonics on Earth (*62*). At this time, light-δ^60^Ni meteoritic material added to Earth’s mantle much earlier, in the early Hadean Eon (immediately after core formation), was thoroughly mixed into almost the whole mantle. Similarly, according to the mantle-source HSE variations of the Archean and Proterozoic komatiites, Puchtel *et al.* (*35*) have calculated that these late-accreted materials (accreted during the first ~150 Ma of the Solar System history) would have been completely homogenized within the mantle by 2.5 ± 0.2 Ga. The observed Ni isotopic difference between Archean ultramafic rocks and post-Archean unmetasomatized mantle peridotites agrees well with the documented near-complete disappearance of resolvable ^142^Nd anomalies and positive ^182^W offsets in the post-Archean mantle (*41*, *63*–*67*). In detail, Hyung and Jacobsen (*67*) further demonstrated the homogenization of ^142^Nd heterogeneity throughout Earth’s history, with a rapid mantle stirring rate of about 400 million years since the early Hadean. Our documenting of temporal changes in Ni isotopic composition in Earth’s early history (Fig. 6) is consistent with the fast mantle stirring rate indicated by the short-lived Sm-Nd isotope system and also hints that Earth’s chemical and isotopic evolution has been largely regulated by plate tectonics since the late Archean. Considering that the predominant tectonic regime in the Hadean was likely stagnant-lid convection (*68*), we infer that the Moon-forming giant impact and the subsequent onset of mobile-lid plate tectonics successively created the multistage evolution of Ni isotopic composition of the terrestrial mantle. A similar temporal variation in Earth’s mantle convective regime to that documented by Nd isotopes and Ni isotopes was recently captured by temporal variability in Ti stable isotopes (*69*). It is therefore entirely possible that mantle convection at the end of the Archean eon was a key factor in controlling the variability in Ni isotopic composition documented by our study.

Mass-dependent Ni isotopic changes throughout the time scale of Earth’s mantle evolution cannot, alone, clearly demonstrate the source of late-accreted materials that contributed isotopically light Ni or the proto-Earth’s original compositions. Nevertheless, our compiled data require that these components were generated early on, before the late veneer witnessed by incomplete HSE accretion and s-process Ru excess, and subsequently further mixed with the proto-Earth mantle driven by plate tectonics. Despite the on-going debate on the origin of Earth’s isotopically light Ni, it is clear that the mantle requires a complex evolutionary history of multistage Ni isotopic changes and the heterogeneous ingrowth of the BSE. The evolution of the different reservoirs of Earth’s mantle can be traced through the combined mass-dependent and mass-independent Ni isotopic systems and provides implications for the mode of convection that may have governed mantle mixing processes, to the suggested timing (e.g., the end of late Archean) of when plate tectonics was initiated.

## MATERIALS AND METHODS

### Geological background and sample description

The >3000-km^2^ IGC (Fig. 1) of Southwest Greenland consists of ~90% TTG gray gneisses and ~10% supracrustal, mafic, and ultramafic rocks, with an age span from ≥3870 to ~3600 million years ago (Ma), preserving an archive of early terrestrial crustal evolution (*70*). The IGC is further divided into two Eoarchean crustal terranes (i.e., Isuakasia and Færingehavn) and some adjacent younger Meso- to Neoarchean terranes (*42*). Compared with several peridotite enclaves and layered intrusions within or near the ISB in the Isuakasia Terrane, the NUB (Fig. 1) emplaced onto the Færingehavn Terrane is less well studied. The NUB is a ca. 1-km-long body of ultramafic rocks associated with meta-gabbros, siliceous rocks, and amphibolites that are enclosed by polyphase TTGs. Minimum ages of these ultramafic rocks are mainly constrained by U-Pb zircon dating of cross-cutting TTG intrusives, yielding an Eoarchean age at 3650 Ma (*43*). Recently, whole-rock Lu-Hf isochrons obtained from the NUB peridotite enclaves also yield an Eoarchean age of 3.93 ± 0.15 Ga (with initial ^176^Hf/^177^Hf = 0.28033 ± 11) (*21*) that is consistent with the inference from field relationship.

The NUB was strongly deformed by amphibolite facies to locally granulite facies metamorphism at ~2.7 Ga (*42*). Most of the NUB ultramafic rocks are highly deformed, altered, and locally pervaded by discordant veins of coarse-grained phlogopite and fibrous amphibole (*21*). The selected 13 relatively fresh NUB samples are meta-peridotites that comprise different proportions of olivine (37 to 90%) + amphibole (0 to 40%) + chromite (2 to 10%) + chlorite (2 to 10%) ± minor serpentine (<3%; table S1 and fig. S9). Three strongly altered samples (chlorite, ~50 to 65%) were also selected for analysis to compare the influence of long-term secondary hydrothermal alteration on Ni isotopes of NUB rocks. Almost all the NUB samples lack visible sulfides, except for a few pentlandites observed in samples 208191 and 208196 (fig. S9). Note that our NUB samples and those reported previously with s-process Ru excess relative to the BSE (*19*) were collected from the same batch, thus making them a unique recorder of Earth’s pre-late-veneer mantle.

### Nickel stable isotopic analysis

High-precision mass-dependent Ni isotope analyses were conducted at the Isotope Geochemistry Laboratory, China University of Geosciences, Beijing (CUGB). About 50 mg of sample powder was digested in distilled HF-HNO_3_ at 200°C and high pressure in polytetrafluoroethylene bombs. After complete digestion, aliquots of sample solutions containing 800 ng of Ni were equilibrated with a ^61^Ni-^62^Ni double spike to reach an optimal ratio of ^62^Ni_spike_/^58^Ni_sample_ ≈ 1.3 (*71*). Nickel was then separated from the matrix using a four-step ion exchange chemistry following that of (*71*). Step 1 column uses AG 50W-X8 and AG 1-X8 resins to remove Fe and Ca. Step 2 column uses AG 50W-X8 resin to separate Ni from Mg, Ti, and Al in a media of 0.15 M HNO_3_ and 4 M HF. Step 3 uses 0.5 M HCl containing 95% acetone to remove Mn, and the last step further separates Ni from the residual matrix using 0.5 M HCl + 95% acetone +0.1 M dimethylglyoxime. The Ni yields of the entire purification procedure were >90%, while the total Ni blanks were lower than 1.2 ng. The Ni isotope ratios were determined on a Neptune Plus MC-ICPMS using a low-resolution mode with an Aridus II introduction system [see detailed cup configuration and instrument performance in (*72*)]. The spiked standard NIST SRM 986 with similar Ni concentrations was repeatedly analyzed before and after every five samples to monitor the long-term stability. Data reduction was performed offline via an Excel worksheet. Isotopic compositions are presented as δ^60^Ni per mil deviations relative to NIST SRM 986

$$\delta^{60}\mathrm{Ni}=\left[\frac{{\left({}^{60}\mathrm{Ni}/{}^{58}\mathrm{Ni}\right)}_{\mathrm{sample}}}{{\left({}^{60}\mathrm{Ni}/{}^{58}\mathrm{Ni}\right)}_{\mathrm{SRM}\;986}}-1\right]\times 1000$$

The long-term precision during various analytical campaigns over the past 2 years, based on replicated analyses of NIST SRM 986, was better than 0.03‰ (2SD, *n* = 128) on the δ^60^Ni value (*73*). Each sample solution was measured at least three times in different analytical sessions, and the average value was reported together with 2SD. The δ^60^Ni of international geological reference materials JP-1 (dunite), BHVO-2 (basalt), and NOD-P-1 (Mn nodule) are 0.14 ± 0.01 (2SD, *n* = 6), 0.04 ± 0.02 (2SD, *n* = 6), and 0.32 ± 0.02 (2SD, *n* = 6), respectively (table S4), all of which are in excellent agreement with their previously reported values (*71*–*73*).

### Bulk-rock major and trace elements, mineral major elements, HSEs, and Re-Os isotopic analysis

Analytical methods for HSEs (here including Re + five PGEs: Os, Ir, Ru, Pt, and Pd) and Re-Os isotopes (analyzed at CUGB) are described in detail in (*34*), while those for some related bulk-rock major and trace elements (analyzed at Washington State University; table S1), as well as mineral (e.g., olivine, amphibole, and chromite) major elements (analyzed at University of Copenhagen; table S3) involved in this work, are described in (*74*). The HSE concentrations and ^187^Os/^188^Os ratios of repeatedly measured reference materials UB-N (serpentinized lherzolite) and BHVO-2 (table S4) are also consistent with their accepted values (*75*) within uncertainties.

### Confidence intervals and statistical analysis

The 95% CI for the sample average was calculated using the following formula, which is based on an estimate of *t* distribution

$$95\%\mathrm{CI}=\bar{X}\pm t_{95}\left(\frac{S_{\bar{X}}}{\sqrt{n}}\right)$$

where $\bar{X}$ is the sample arithmetic mean, *t*_95_ is the *t* value for a two-tailed test with an α value of 0.05, $S_{\bar{X}}$ is the sample SD, and $\sqrt{n}$ is the square root of the sample size (*n*). Notably, *t*_95_ relies on the degrees of freedom (df *= n* − 1) as a reflection of sample size. With an infinitely large sample size, the *t* distribution and the standard normal distribution will be the same, and for samples greater than 30, they will be very similar (e.g., the unmetasomatized post-Archean mantle peridotites with *n* = 40), but the *t* distribution will be somewhat more conservative. Therefore, to relatively small sample size data populations, we can reasonably apply a *t* distribution instead of the standard normal distribution to compute the 95% CI, as a conservative measure of the uncertainty around a central value (the mean).

Given that the sample size is small, two statistical tests, including the Welch’s *t* test and Mann-Whitney *U* test, were conducted to statistically test whether the mass-dependent Ni isotopic compositions for the 3.8-Ga NUB cumulates (*n* = 10), the modern BSE (constrained by unmetasomatized post-Archean mantle peridotites, *n* = 40), and the chondritic reservoirs (25 carbonaceous + 18 ordinary + 13 enstatite chondrites) are identical or not. The Welch’s *t* test, also known as unequal variances *t* test, is generally applied when there is a difference between the variations of two populations and also when their sample sizes are unequal. The Mann-Whitney *U* test is a nonparametric version of the independent samples *t* test and primarily assesses the difference between two sample groups with low numbers of individuals in each group (usually less than 30), which are not normally distributed. Taking the comparison between NUB and BSE datasets as an example, the designated null hypothesis (*H*_0_) is that the average δ^60^Ni values of both populations are equal. If the two-tailed *P* value is less than the defined significance threshold (α) of 0.05, then the *H*_0_ can be rejected at 95% confidence. We find that both the Welch’s *t* test (*P <* 0.00001) and Mann-Whitney *U* test (*P =* 0.00012) yield two-tailed *P* values <<0.05, indicating that we can confidently reject the null hypothesis (*H*_0_) and conclude that the 3.8-Ga NUB cumulates (mean δ^60^Ni = 0.16 ± 0.01‰) are isotopically heavier than the post-Archean mantle peridotites (mean δ^60^Ni = 0.11 ± 0.01‰). Similarly, according to the comparison via the Welch’s *t* test (*P* < 0.00001) and Mann-Whitney *U* test (*P* < 0.00001), we can claim that the observed Ni isotopic difference between the NUB cumulates and chondritic reservoirs (mean δ^60^Ni = 0.23 ± 0.02‰) is statistically significant at 95% confidence.

## Supplementary Materials

### This PDF file includes:

Figs. S1 to S12 Tables S1 to S5 References
