# Supplementary material for: Ni isotopes provide a glimpse of Earth’s pre-late-veneer mantle
Source: Sci Adv. 2023 Dec 15;9(50):eadj2170. doi: 10.1126/sciadv.adj2170 (PMC11649070; doi:10.1126/sciadv.adj2170)
Supplement: Supplementary file 1 — Figs. S1 to S12 Tables S1 to S5 References [file sciadv.adj2170_sm.pdf]

Science Advances
AAAS

Supplementary Materials for

Ni isotopes provide a glimpse of Earth's pre-late-veneer mantle

Yong Xu *et al.*

Corresponding author: Jingao Liu, jingao@cugb.edu.cn

Sci. Adv. **9**, eadj2170 (2023)
DOI: 10.1126/sciadv.adj2170

This PDF file includes:

Figs. S1 to S12
Tables S1 to S5
References

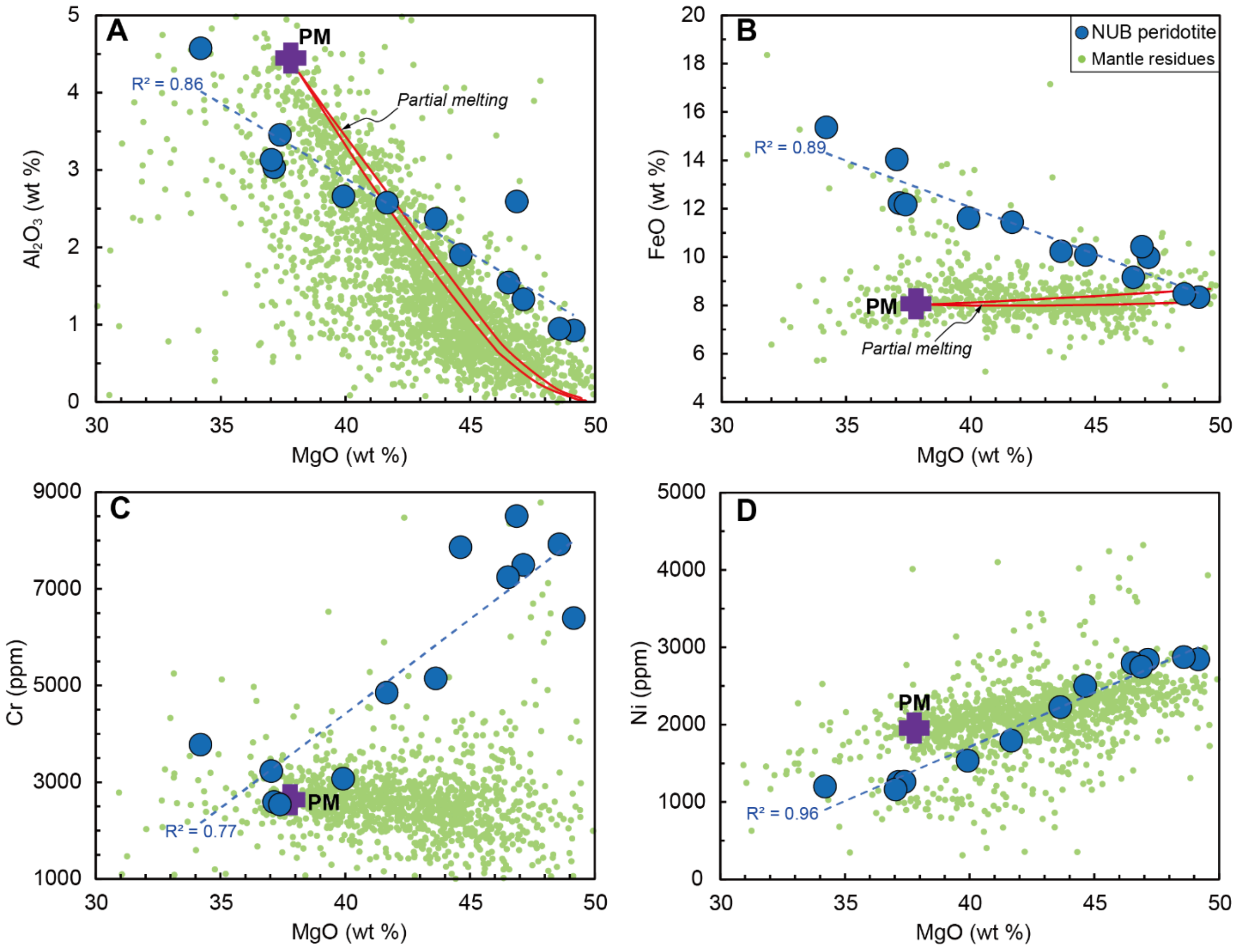

Fig. S1.

Bulk-rock anhydrous major and minor element variations of the fresh NUB peridotites, Al_2O_3 (**A**), FeO (**B**), Cr (**C**), and Ni (**D**) versus MgO concentrations, compared to the Primitive Mantle (PM) (*76*). Mantle residues represented by mantle tectonites and peridotite xenoliths collected from different tectonic settings are also shown for comparison. Data sources of mantle residues are from the PetDB database (www.earthchem.org/petdb). Melting trends are modeled by decompression (2–0, 3–0.2 GPa) fractional partial melting, using the approach of (*81*).

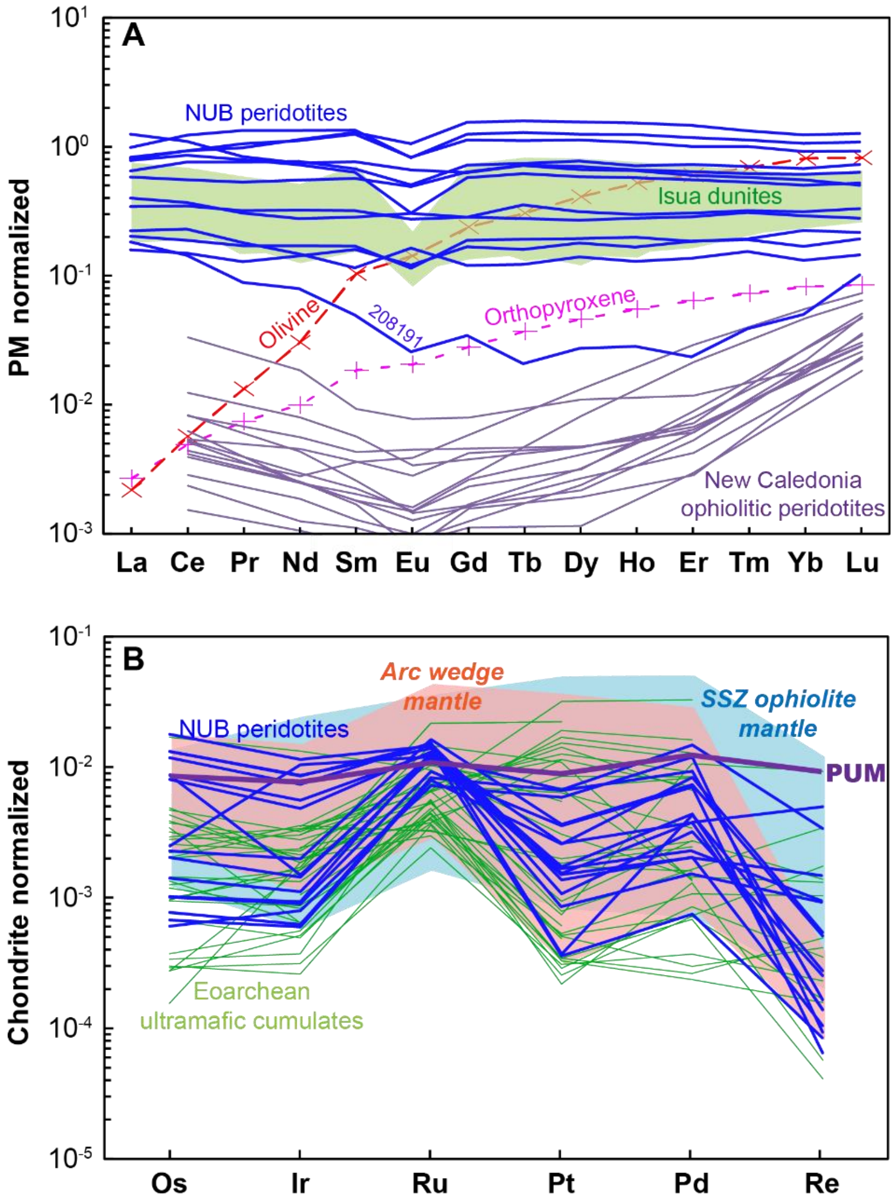

Fig. S2.

Diagrams of rare-earth-element (REE) and highly siderophile element (HSE) distribution patterns. (**A**) PM (*82*)-normalized REE patterns for the NUB peridotites, compared to ranges of the Isua dunites (light green field) (*22*), and New Caledonia ophiolite mantle residues (*26*); (**B**) Chondrite (*28*)-normalized HSE patterns for the NUB peridotites (this study and (*6*)), compared to Primitive Upper Mantle (PUM) estimate (*29*). Light pink and blue shaded fields are ranges for mantle peridotites from arc wedges and SSZ ophiolites, respectively (after (*22*)). Green thin lines represent Eoarchean ultramafic cumulates collected from the literature (*22*–*24*).

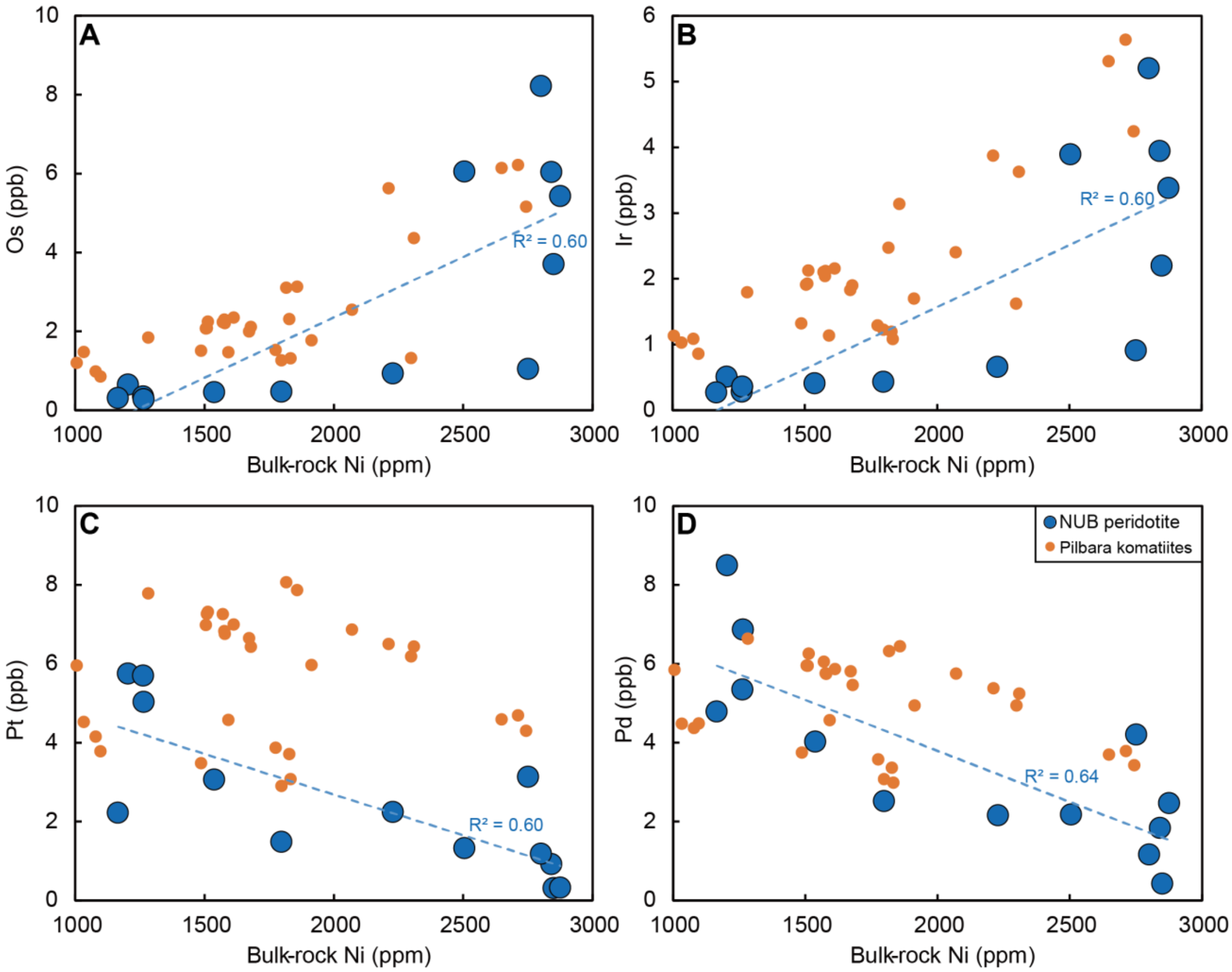

Fig. S3.

Bulk-rock Os (**A**), Ir (**B**), Pt (**C**), and Pd (**D**) abundances versus Ni concentrations of the fresh NUB peridotites.

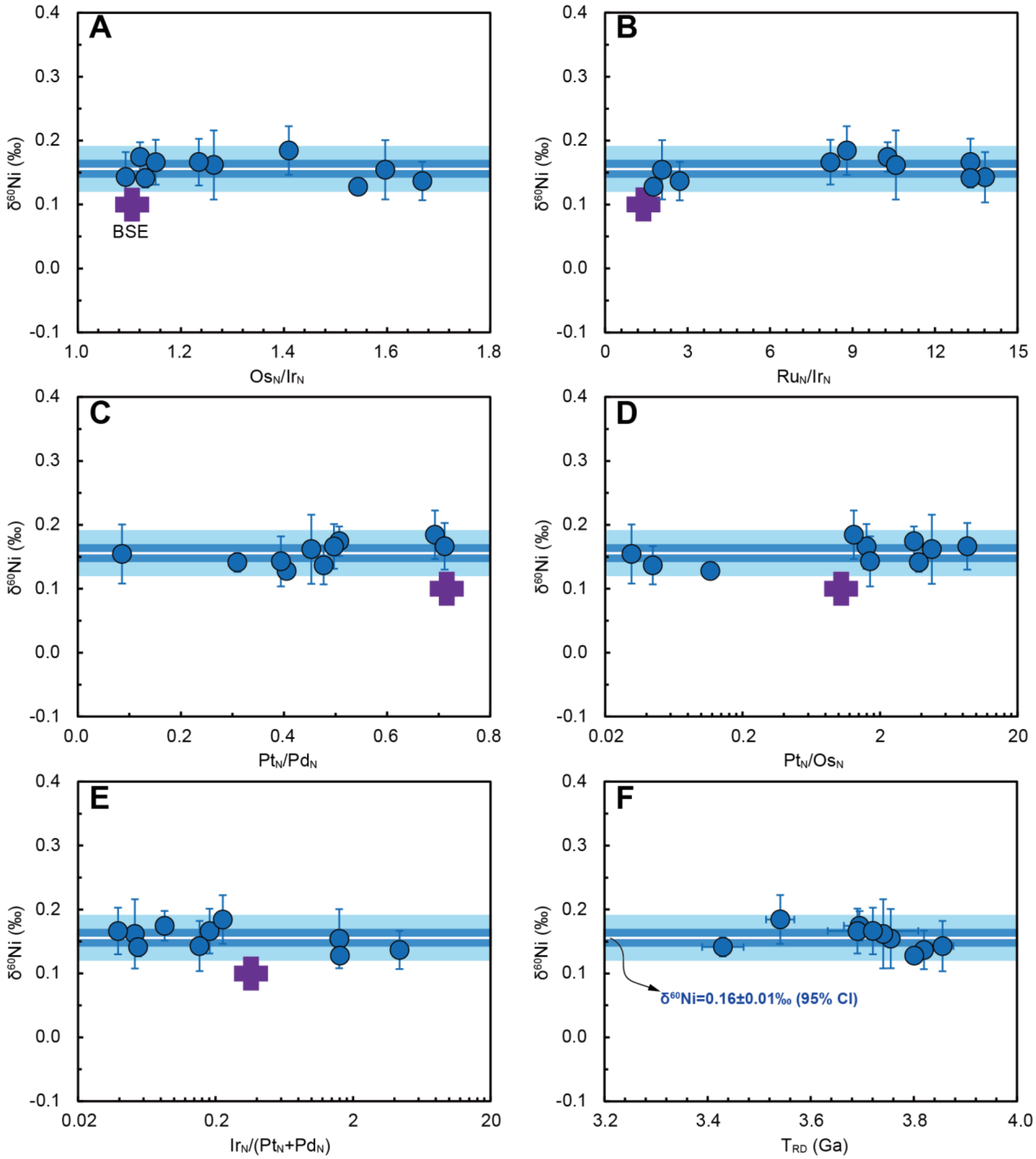

Fig. S4.

Bulk-rock δ^{60}Ni values versus Os_N/Ir_N (**A**), Ru_N/Ir_N (**B**), Pt_N/Pd_N (**C**), Pt_N/Os_N (**D**) and $Ir_N/(Pt_N+Pd_N)$ (**E**) ratios, and T_{RD} ages (**F**) for the fresh NUB peridotites. The 'N' represents chondrite-normalized values. Light and dark blue shaded fields represent the 2SD (±0.03‰) and 95% confidence interval (±0.01‰) for the NUB sample group, respectively.

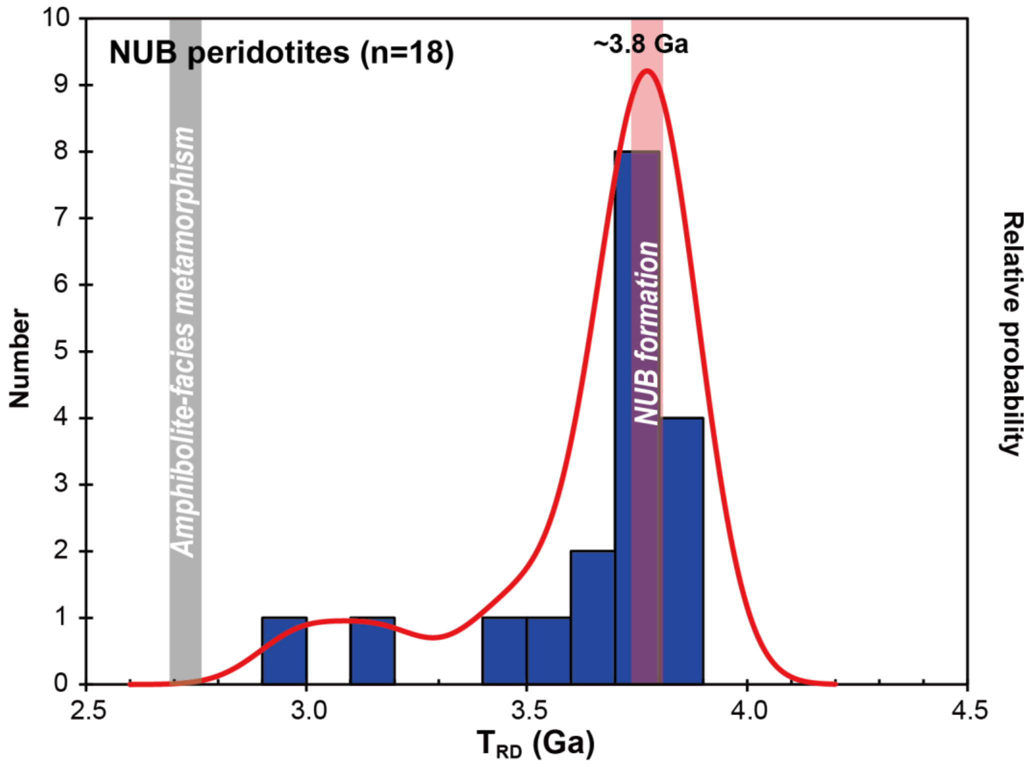

Fig. S5.

Histograms and kernel density estimation (KDE) plots of T_{RD} ages of the NUB peridotites (15 data from this study and 3 data from (*6*)). An uncertainty of 0.2 Ga (2SE) was assumed for all single data points.

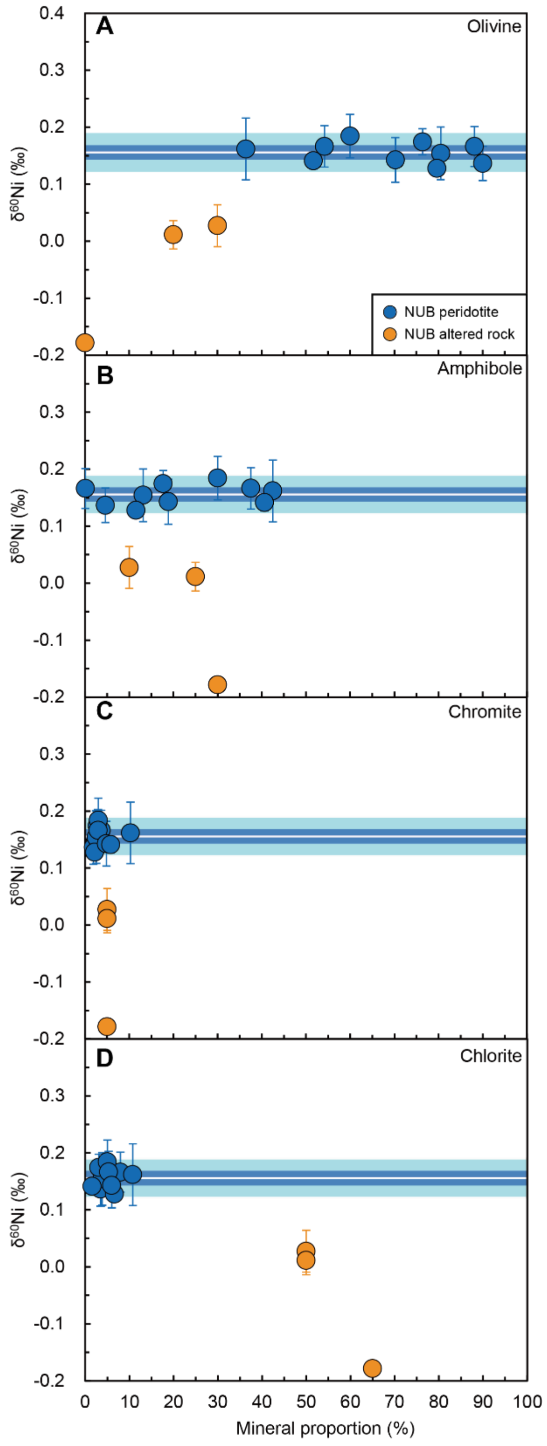

Fig. S6.

Bulk-rock δ^{60}Ni values versus modal olivine (**A**), amphibole (**B**), chromite (**C**), and chlorite (**D**) abundances for the ~3.8 Ga NUB fresh peridotites and altered rocks. Light and dark blue shaded fields represent the 2SD (±0.03‰) and 95% confidence interval (±0.01‰) for the NUB sample group, respectively.

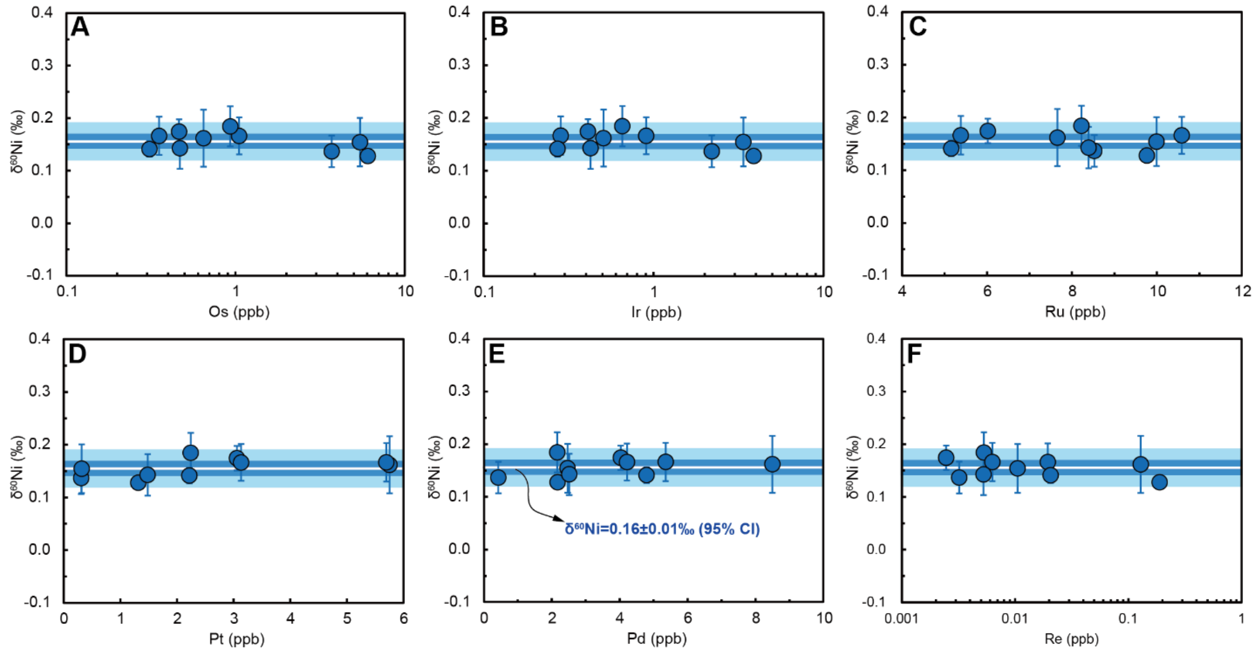

Fig. S7.

Bulk-rock $\delta^{60}Ni$ values versus Os (**A**), Ir (**B**), Ru (**C**), Pt (**D**), Pd (**E**) and Re (**F**) abundances for the fresh NUB peridotites. Light and dark blue shaded fields represent the 2SD (±0.03‰) and 95% confidence interval (±0.01‰) for the NUB sample group, respectively.

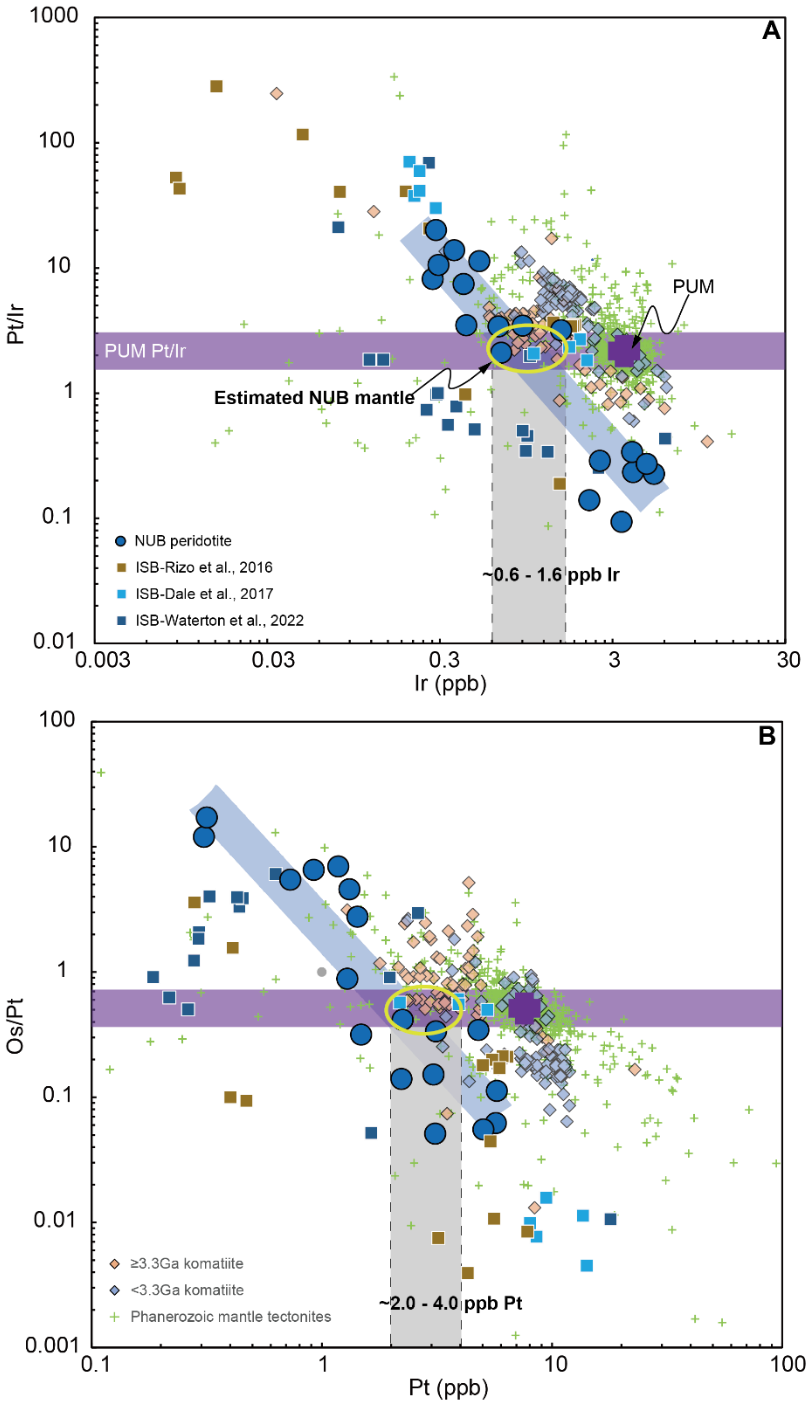

Fig. S8.

Ratios of moderately compatible HSE (Pt) to highly compatible HSE (Os-(**A**), Ir-(**B**)), plotted against the denominator. Data of ⩾3.3 Ga and <3.3 Ga komatiites, Phanerozoic mantle tectonites, and ISB (Isua Supracrustal Belt) ultramafic rocks are also shown for illustration. Data sources are the same as Fig. 5 in the main text.

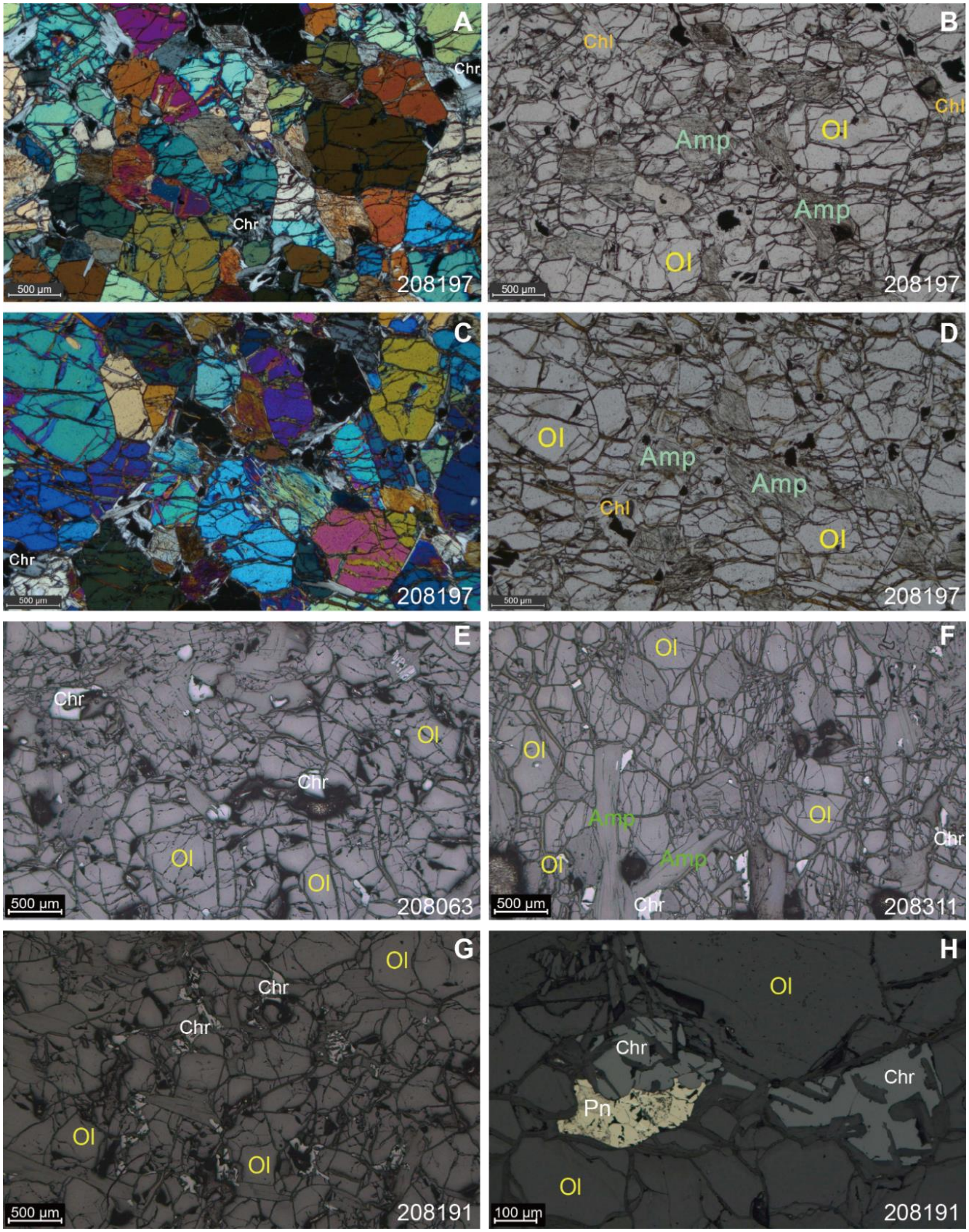

Fig. S9.

Transmission (**A-D**) and reflected (**E-H**) light electronic micrographs of the fresh NUB peridotites. Mineral abbreviation: Ol, olivine; Amp, amphibole; Chl, chlorite; Chr, chromite; Pn, pentlandite.

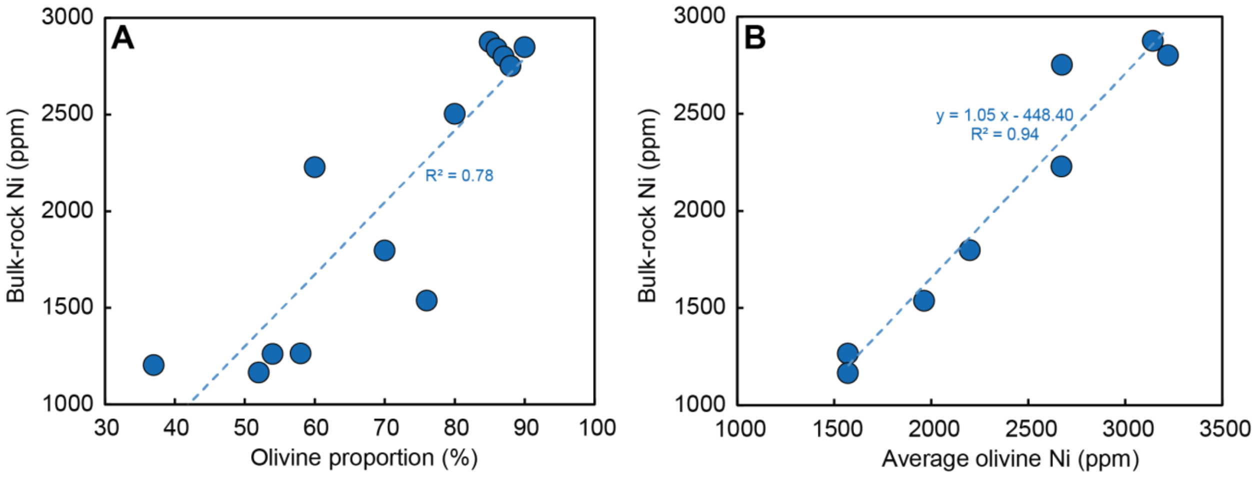

Fig. S10.

Bulk-rock Ni concentrations versus olivine proportions (**A**) and average olivine Ni contents (**B**) of the fresh NUB peridotites.

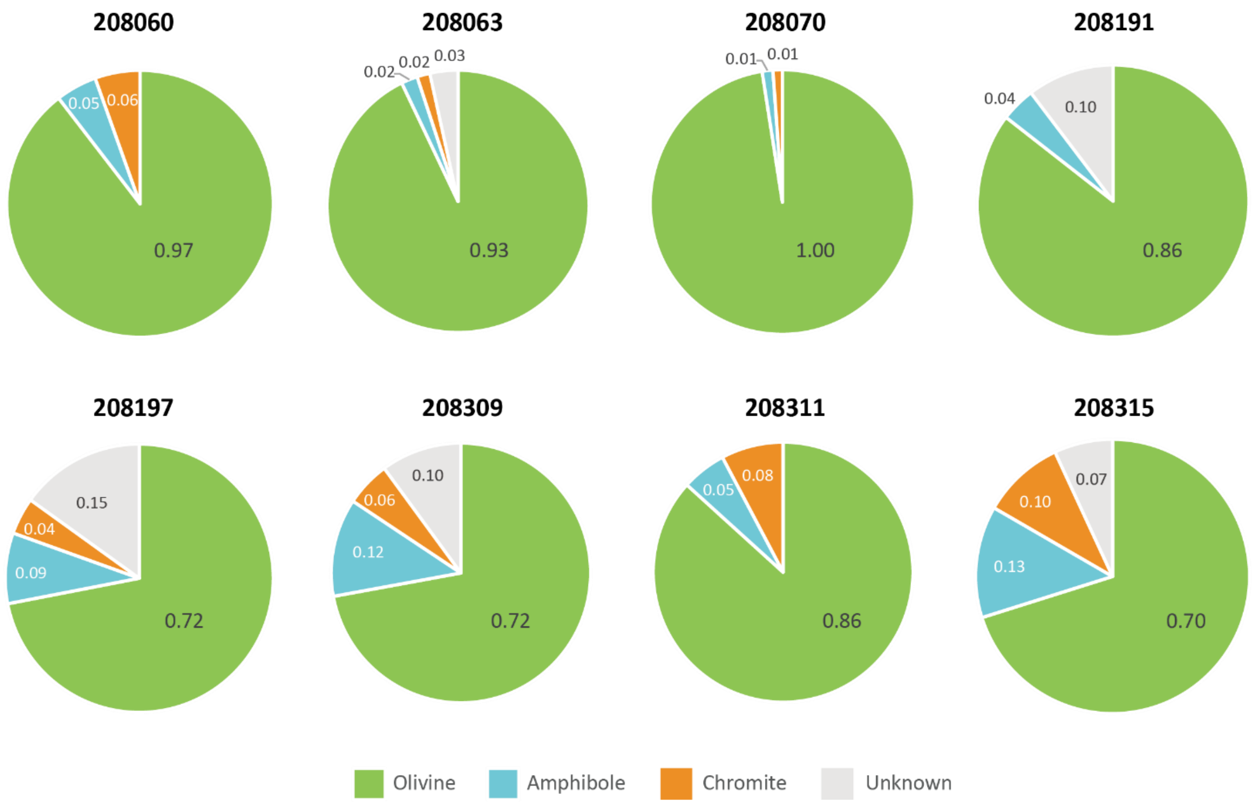

Fig. S11.

The pie charts of the Ni budget proportions of major minerals (e.g., olivine, amphibole, chromite) accounting for the bulk-rock Ni concentrations of the fresh NUB peridotites. Due to analytical errors in model mineral estimates and EPMA measurements, the calculated total proportions of 208060 and 208070 slightly exceed 100%, but it does not affect the assessment of the contribution of each mineral to the bulk Ni budget.

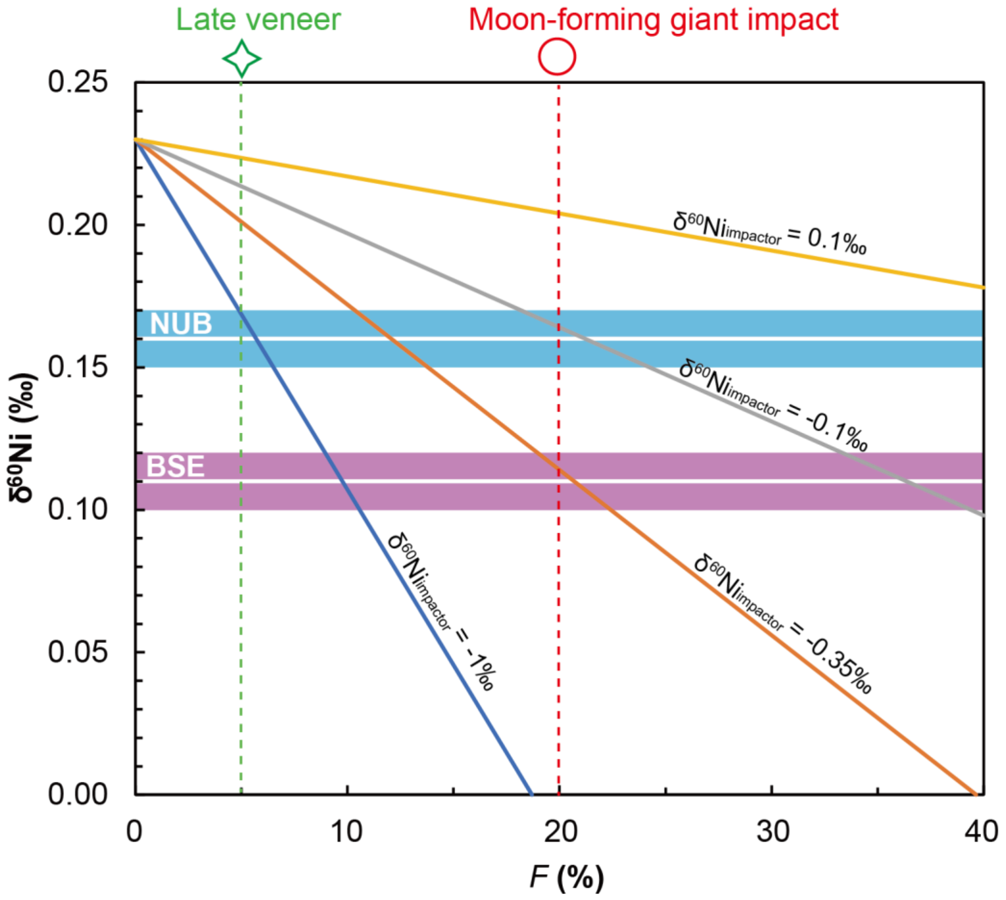

Fig. S12.

Mass balance model estimates the influence of late-stage impactors (with varied δ^{60}Ni values of 1.0‰, -0.35‰, -0.1‰ and +0.1‰) on the Ni isotopic composition of the proto-BSE with an assumed chondrite-like δ^{60}Ni value of 0.23‰, modified after (*10*). The '*F*' represents the contribution extent of different late accretion events to Ni of the bulk silicate earth (BSE). Accretion models proposed by (*83*) imply that the Moon-forming impactor (Theia) contributed ~20% of Ni (red circle) and the late veneer contributed less than 5% (green star).

Table S1.

Mineral modal proportions (%), bulk-rock major (in wt %) and trace element (ppm) concentrations, HSE abundances (ppb) and Re-Os isotopic compositions of the Eoarchean Narssaq ultramafic rocks, Southwest Greenland.

Sample	208060	208062	208063	208064	208066	208070	208191	208196	208197	208305	208309	208311	208315	208190	208194	209321
Olivine (%)	76	90	85	86	80	87	88	37	60	54	58	70	52	35	20	
Amphibole	17	5	8	5	12	5		40	30	38	34	19	40	10	25	30
Chlorite	2	3	3	4	4	4	6	10	5	3	3	4	2	50	50	65
Chromite	3	2	3	3	2	3	4	10	3	3	3	5	5	2	3	5
Serpentine	2		1	2	2	1	2	3	2	2	2	2	1	3	2	
SiO_2 (wt %)	43.25	40.83	41.18	40.71	41.84	41.66	39.88	41.04	41.71	42.92	42.67	42.22	41.28	41.36	43.72	38.11
TiO_2	0.12	0.04	0.04	0.06	0.08	0.06	0.08	0.29	0.10	0.19	0.15	0.11	0.13	0.31	0.33	0.57
Al_2O_3	2.66	0.93	0.95	1.33	1.91	1.54	2.59	4.57	2.37	3.03	3.46	2.58	3.13	10.52	5.90	15.31
FeO	11.61	8.34	8.47	10.00	10.08	9.17	10.43	15.36	10.23	12.24	12.17	11.44	14.04	5.76	10.60	10.29
MnO	0.15	0.14	0.15	0.16	0.18	0.15	0.14	0.17	0.15	0.16	0.19	0.16	0.19	0.07	0.15	0.05
MgO	39.92	49.16	48.58	47.13	44.62	46.53	46.87	34.20	43.63	37.14	37.38	41.67	37.03	40.17	33.13	32.30
CaO	2.21	0.52	0.64	0.60	1.27	0.87	0.00	3.98	1.74	3.92	3.61	1.78	3.78	1.67	5.39	3.23
Na_2O	0.02	0.00	0.00	0.00	0.00	0.00	0.00	0.34	0.00	0.34	0.31	0.00	0.37	0.00	0.64	0.09
K_2O	0.04	0.05	0.00	0.00	0.00	0.02	0.00	0.04	0.06	0.04	0.05	0.04	0.04	0.14	0.11	0.05
P_2O_5	0.01	0.00	0.00	0.00	0.01	0.00	0.01	0.01	0.01	0.01	0.01	0.01	0.01	0.01	0.03	0.01
LOI	2.44	4.13	1.82	1.94	3.13	3.25	2.35	2.17	2.37	2.10	1.81	2.89	2.63	7.91	3.22	8.59
Bulk Mg#	86.0	91.3	91.1	89.4	88.7	90.0	88.9	79.9	88.4	84.4	84.6	86.7	82.5	92.6	84.8	84.8
Ni (ppm)	1537	2849	2875	2841	2504	2800	2751	1204	2228	1262	1264	1797	1165	1834	934	989

Cr	3073	6396	7923	7499	7861	7243	8508	3780	5151	2595	2542	4857	3234	5517	1372	2770
La	0.849	0.151	0.107	0.136	0.232	0.270	0.123	0.560	0.530	0.669	0.546	0.395	0.440	0.512	0.972	0.339
Ce	1.930	0.402	0.261	0.328	0.608	0.645	0.251	1.628	1.505	2.154	1.622	0.971	1.329	1.306	2.986	0.905
Pr	0.228	0.049	0.035	0.046	0.087	0.083	0.024	0.267	0.215	0.363	0.288	0.144	0.207	0.169	0.505	0.158
Nd	0.995	0.197	0.190	0.227	0.432	0.371	0.106	1.512	0.964	1.789	1.490	0.735	1.026	0.739	2.773	0.853
Sm	0.335	0.050	0.070	0.074	0.139	0.124	0.022	0.566	0.277	0.591	0.548	0.248	0.295	0.158	0.932	0.331
Eu	0.110	0.027	0.020	0.019	0.045	0.050	0.004	0.175	0.051	0.137	0.137	0.081	0.084	0.081	0.267	0.134
Gd	0.369	0.070	0.098	0.110	0.167	0.167	0.020	0.911	0.338	0.734	0.661	0.374	0.422	0.202	1.351	0.423
Tb	0.076	0.013	0.017	0.020	0.038	0.030	0.002	0.168	0.066	0.138	0.119	0.077	0.079	0.034	0.250	0.078
Dy	0.485	0.102	0.130	0.141	0.227	0.195	0.020	1.130	0.424	0.908	0.819	0.511	0.561	0.215	1.574	0.496
Ho	0.105	0.021	0.027	0.032	0.048	0.045	0.005	0.248	0.093	0.201	0.175	0.107	0.115	0.046	0.343	0.110
Er	0.283	0.064	0.087	0.087	0.145	0.135	0.011	0.692	0.262	0.556	0.476	0.303	0.344	0.142	0.963	0.304
Tm	0.042	0.011	0.014	0.014	0.023	0.023	0.003	0.097	0.039	0.083	0.073	0.045	0.051	0.021	0.122	0.041
Yb	0.270	0.064	0.082	0.109	0.152	0.142	0.024	0.601	0.245	0.516	0.447	0.296	0.328	0.129	0.792	0.271
Lu	0.037	0.011	0.014	0.016	0.024	0.020	0.007	0.092	0.038	0.079	0.067	0.046	0.053	0.022	0.116	0.040
Os (ppb)	0.46	3.70	5.43	6.04	6.05	8.22	1.05	0.65	0.93	0.35	0.28	0.47	0.31	1.65	0.16	0.66
Ir	0.41	2.20	3.38	3.94	3.89	5.20	0.91	0.51	0.66	0.28	0.36	0.43	0.27	1.51	0.29	0.45
Ru	6.02	8.52	9.99	9.01	9.76	9.41	10.58	7.66	8.22	5.38	4.70	8.39	5.16	8.39	3.03	5.49
Pt	3.06	0.31	0.32	0.92	1.32	1.18	3.13	5.75	2.24	5.70	5.03	1.48	2.22	4.79	3.11	1.32
Pd	4.03	0.43	2.47	1.84	2.18	1.17	4.21	8.50	2.16	5.35	6.87	2.52	4.79	2.17	5.51	3.36
Re	0.002	0.003	0.011	0.010	0.189	0.004	0.019	0.129	0.005	0.006	0.004	0.005	0.021	0.015	0.003	0.008
$^{187}Re/^{188}Os$	0.025	0.004	0.009	0.008	0.150	0.002	0.089	0.954	0.027	0.085	0.061	0.054	0.318	0.045	0.093	0.055
2SE	0.004	0.000	0.000	0.000	0.008	0.000	0.003	0.044	0.001	0.005	0.005	0.003	0.014	0.002	0.007	0.003

$^{187}Os/^{188}Os$	0.10211	0.10115	0.10165	0.10116	0.10130	0.10115	0.10214	0.10176	0.10329	0.10191	0.10750	0.10087	0.10414	0.10132	0.10608	0.10262
2SE	0.00022	0.00007	0.00008	0.00017	0.00007	0.00007	0.00009	0.00014	0.00020	0.00063	0.00020	0.00015	0.00029	0.00009	0.00037	0.00017
Initial $^{187}Os/^{188}Os$†	0.1004	0.1009	0.1010	0.1007	0.0915	0.1010	0.0963	0.0394	0.1015	0.0963	0.1035	0.0974	0.0833	0.0984	0.1000	0.0990
T_{RD} (Ga) †	3.69	3.82	3.76	3.82	3.80	3.82	3.69	3.74	3.54	3.72	2.99	3.86	3.43	3.80	3.18	3.63
T_{MA} (Ga) †	3.92	3.86	3.83	3.89	5.72	3.84	4.61	-3.30	3.77	4.60	3.47	4.38	12.04	4.22	4.02	4.14

*Bulk-rock Mg# = atomic $Mg^{2+}/[Mg^{2+}+Fe^{2+}]\times100$;

†Re-Os model ages (T_{MA} and T_{RD}) are calculated using the Primitive Upper Mantle (PUM) reference ($^{187}Os/^{188}Os = 0.1296$, $^{187}Re/^{188}Os = 0.433$) (*30*), along with $\lambda_{Re} = 1.666 \times 10^{-11}$/year (*84*). An average age of 3.8 Ga for the Itsaq gneiss complex (IGC; (*70*)) is applied for initial $^{187}Os/^{188}Os$ calculation.

Table S2.

Mass-dependent nickel stable isotopic compositions (expressed as $\delta^{60}Ni$, ‰) for the Eoarchean Narssaq ultramafic rocks, Southwest Greenland.

Sample	Lithology	$\delta^{60}Ni$	2SD	n
208060	Peridotite	0.17	0.02	3
208062	Peridotite	0.14	0.03	3
208063	Peridotite	0.15	0.04	3
208066	Peridotite	0.13	0.01	3
208191	Peridotite	0.17	0.04	3
208196	Peridotite	0.16	0.04	3
208197	Peridotite	0.18	0.04	3
208305	Peridotite	0.17	0.04	3
208311	Peridotite	0.14	0.04	3
208315	Peridotite	0.14	0.01	3
208190	Altered	0.03	0.04	3
208194	Altered	0.01	0.02	3
209321	Altered	-0.18	0.01	3

Table S3.

Olivine, amphibole and chromite/spinel average major element compositions (in wt %) of the Eoarchean Narssaq ultramafic rocks, Southwest Greenland.

Sample	SiO_2	TiO_2	Cr_2O_3	Al_2O_3	FeO	MnO	MgO	NiO	CaO	K_2O	Na_2O	Total	Fo*
Olivine													
208060	40.10	0.01	0.01	0.00	10.96	0.17	47.23	0.25	0.00	0.01	0.01	98.75	88.48
208063	41.16	0.01	0.01	0.00	8.05	0.15	50.34	0.40	0.01	0.01	0.05	100.19	91.77
208070	41.04	0.01	0.01	0.00	9.14	0.15	49.61	0.41	0.01	0.01	0.05	100.44	90.63
208191	41.25	0.01	0.01	0.01	9.60	0.15	49.45	0.34	0.00	0.01	0.01	100.83	90.18
208197	40.75	0.01	0.01	0.01	10.51	0.18	48.97	0.34	0.00	0.01	0.01	100.80	89.25
208309	41.24	0.01	0.01	0.01	12.26	0.25	47.65	0.20	0.00	0.01	0.01	101.64	87.39
208311	41.05	0.01	0.00	0.00	11.23	0.18	47.62	0.28	0.00	0.01	0.02	100.41	88.32
208315	41.20	0.01	0.00	0.01	11.30	0.26	47.90	0.20	0.01	0.00	0.01	100.90	88.31
Amphibole													
208060	55.66	0.11	0.12	1.99	6.74	0.22	26.27	0.06	4.23	0.13	0.41	95.94	
208063	55.56	0.22	0.38	2.74	2.01	0.07	22.52	0.09	12.57	0.07	0.65	96.88	
208070	52.55	0.46	0.71	5.43	2.59	0.07	21.38	0.10	12.68	0.11	1.23	97.31	
208197	53.54	0.30	0.55	4.32	3.10	0.08	21.91	0.08	12.03	0.18	0.95	97.03	
208309	53.71	0.23	0.20	4.89	3.80	0.11	21.36	0.06	12.51	0.17	1.02	98.05	
208311	54.85	0.21	0.32	3.28	4.57	0.16	23.04	0.07	9.84	0.12	0.81	97.27	
208315	50.93	0.20	0.27	7.15	4.06	0.07	20.10	0.05	12.93	0.15	1.45	97.35	
Chromite or Fe oxides													
208060	0.09	0.30	9.48	0.87	78.72	0.14	0.93	0.39	0.00	0.01	0.02	90.95	

208063	0.03	0.89	46.02	8.45	38.81	0.63	5.12	0.20	0.00	0.01	0.02	100.17
208070	0.05	0.66	43.95	13.58	35.09	0.59	5.74	0.14	0.00	0.01	0.03	99.84
208191	0.04	0.94	30.41	1.09	61.34	0.48	2.07	0.36	0.00	0.00	0.02	96.74
208197	0.04	0.69	24.10	1.51	66.67	0.45	1.48	0.42	0.00	0.01	0.03	95.41
208309	0.19	1.77	7.53	1.01	82.59	0.22	1.42	0.30	0.00	0.01	0.04	95.08
208311	0.03	4.75	18.32	1.14	67.38	0.40	1.87	0.35	0.00	0.00	0.05	94.31
208315	0.03	0.66	5.95	0.99	84.57	0.24	1.21	0.29	0.00	0.01	0.02	93.96

*Fo: Olivine Mg# = atomic $Mg^{2+}/[Mg^{2+}+Fe^{2+}]\times100$.

Table S4.

Mass-dependent nickel stable isotopic compositions (expressed as $\delta^{60}Ni$, ‰), HSE concentrations (ppb) and $^{187}Os/^{188}Os$ ratios of the repeatedly measured international reference materials.

Sample	Lithology	$\delta^{60}Ni$	2SD	n	Os	Ir	Ru	Pt	Pd	Re	$^{187}Os/^{188}Os$	2SE	n
JP-1	Dunite	0.15	0.01	3									
	Replicate	0.14	0.01	3									
	Ave.	0.14	0.01										
UB-N	Lherzolite				3.71	3.34	6.97	7.44	5.93	0.210	0.1274	0.0001	3
BHVO-2	Basalt	0.04	0.02	6	0.12	0.07	0.11	9.05	2.01	0.558	0.1427	0.0002	3
NOD-P-1	Mn nodule	0.32	0.02	6									

Table S5.

Data sources for the age and $\delta^{60}Ni$ of the collected samples shown in Fig. 6.

Location	Sample	Rock type	Metasomatism	Age (Ga)	Data source for the age	$\delta^{60}Ni$	Data source for the $\delta^{60}Ni$
Narssaq	208062	Ultramafic cumulate		3.8		0.14	
Narssaq	208063	Ultramafic cumulate		3.8		0.15	
Narssaq	208066	Ultramafic cumulate		3.8		0.13	
Narssaq	208191	Ultramafic cumulate		3.8		0.17	
Narssaq	208060	Ultramafic cumulate		3.8	*This study*	0.17	*This study*
Narssaq	208196	Ultramafic cumulate		3.8		0.16	
Narssaq	208197	Ultramafic cumulate		3.8		0.18	
Narssaq	208305	Ultramafic cumulate		3.8		0.17	
Narssaq	208311	Ultramafic cumulate		3.8		0.14	
Narssaq	208315	Ultramafic cumulate		3.8		0.14	
Komati, Barberton	SC5	Komatiite		3.5		0.10	
Komati, Barberton	SC7	Komatiite		3.5		0.16	
Komati, Barberton	SC9	Komatiite		3.5		0.11	
Komati, Barberton	SC15	Komatiite		3.5		0.14	
Hooggenoeg, Barberton	HG5	Komatiite		3.5	(*38*)	0.18	(*10*)
Hooggenoeg, Barberton	HG15	Komatiite		3.5		0.11	
Hooggenoeg, Barberton	HG16	Komatiite		3.5		0.07	
Sandspruit, Barberton	SS1	Komatiite		3.5		0.02	
Sandspruit, Barberton	SS5	Komatiite		3.5		0.09	

Sandspruit, Barberton	SS6	Komatiite		3.5		0.13	
Sandspruit, Barberton	SS8	Komatiite		3.5		0.20	
Alexo, Ontario	A-2	Komatiite		2.7		0.14	
Munro Township, Ontario	m-1	Komatiite		2.7	(*85*)	0.12	(*86*)
Thompson Manitoba	P-2	Komatiite		2.7		0.16	
Alexo, Ontario	Kal-1	Komatiite		2.7		0.17	
Kuandian	HY1-01	harzburgite	yes	2.3		0.12	
Kuandian	HY2-01	lherzolite	yes	2.3		0.14	
Kuandian	HY2-02	lherzolite	no	2.3		0.09	
Kuandian	HY2-03	lherzolite	no	2.3		0.13	
Kuandian	HY2-04	lherzolite	no	2.3	(*87*)	0.07	(*10*)
Kuandian	HY2-05	harzburgite	yes	2.3		0.11	
Kuandian	HY2-06	lherzolite	yes	2.3		-0.08	
Kuandian	HY2-07	lherzolite	no	2.3		0.11	
Kuandian	HY2-14	lherzolite	no	2.3		0.04	
Kuandian	HY2-29	lherzolite	no	2.3		0.09	
Fansi	FS-01	lherzolite	yes	1.8		0.07	
Fansi	FS-04	lherzolite	yes	1.8		0.20	
Fansi	FS-18	harzburgite	yes	1.8		0.12	
Fansi	FS-36	lherzolite	yes	1.8	(*88*)	0.07	(*10*)
Fansi	FS-44	lherzolite	yes	1.8		0.05	
Fansi	FS-45	lherzolite	yes	1.8		0.10	
Fansi	FS-50	lherzolite	yes	1.8		0.13	

Fansi	FS-64	lherzolite	yes	1.8		0.17	
Fansi	FS2-04	harzburgite	yes	1.8		0.16	
Damaping	DMP-04	lherzolite	no	1.8		0.05	
Damaping	DMP-60	lherzolite	yes	1.8		0.10	
Yangyuan	YY-22	lherzolite	yes	1.8		0.01	
Yangyuan	YY-27	lherzolite	yes	1.8		0.01	
Yangyuan	YY-51	harzburgite	yes	1.8		-0.06	
Yangyuan	YY-52	lherzolite	yes	1.8		0.15	
Turon de Tecouere	TT16	lherzolite	no	2.3		0.08	
Turon de Tecouere	TT24	lherzolite	no	2.3		0.14	
Turon de Tecouere	TT27	lherzolite	no	2.3		0.12	
Turon de Tecouere	TT02	lherzolite	no	2.3	(*89*)	0.10	(*10*)
Turon de Tecouere	TT11	lherzolite	no	2.3		0.12	
Turon de Tecouere	TT12	lherzolite	no	2.3		0.11	
Turon de Tecouere	TT13	lherzolite	no	2.3		0.14	
Turon de Tecouere	TT21	lherzolite	no	2.3		0.15	
Horoman	BZ116	harzburgite	no	1.0		0.11	
Horoman	BZ117	harzburgite	no	1.0		0.15	
Horoman	BZ131	harzburgite	no	1.0		0.12	
Horoman	BZ216	harzburgite	no	1.0	(*90*)	0.11	(*11*)
Horoman	BZ203	lherzolite	no	1.0		0.11	
Horoman	BZ134	lherzolite	no	1.0		0.13	
Horoman	BZ143	lherzolite	no	1.0		0.07	

Horoman	BZ251	lherzolite	no	1.0		0.08	
Horoman	BZ252	lherzolite	no	1.0		0.12	
Vitim	312-102	lherzolite	no	2.0		0.13	
Vitim	313-105	lherzolite	no	2.0		0.15	
Vitim	313-240	lherzolite	no	2.0	(*91*)	0.13	(*11*)
Vitim	314-56	lherzolite	no	2.0		0.10	
Vitim	314-58	lherzolite	no	2.0		0.10	
Zabargad	BZ-26	lherzolite	no	1.4		0.13	
Zabargad	BZ-29	lherzolite	no	1.4	(*92*)	0.13	(*11*)
Zabargad	BZ-230	lherzolite	yes	1.4		0.22	
Zabargad	BZ-241	harzburgite	no	1.4		0.04	
Tariat	S-1	lherzolite	no	1.4		0.12	
Tariat	S-2	lherzolite	no	1.4		0.12	
Tariat	S-16	lherzolite	yes	1.4	(*93*)	0.11	(*11*)
Tariat	s-62	harzburgite	yes	1.4		0.15	
Tariat	H-25	harzburgite	yes	1.4		0.15	
Tariat	4230/16	lherzolite	yes	1.4		0.16	

REFERENCES AND NOTES

1. A. N. Halliday, R. M. Canup, The accretion of planet Earth. *Nat. Rev. Earth Environ.* **4**, 19–35 (2023).

2. C.-L. Chou, Fractionation of siderophile elements in the Earth's upper mantle. *Proc. Lunar Planet. Sci. Conf.* **1**, 219–230 (1978).

3. R. J. Walker, K. Bermingham, J. Liu, I. S. Puchtel, M. Touboul, E. A. Worsham, In search of late-stage planetary building blocks. *Chem. Geol.* **411**, 125–142 (2015).

4. N. Dauphas, The isotopic nature of the Earth's accreting material through time. *Nature* **541**, 521–524 (2017).

5. J. M. D. Day, A. D. Brandon, R. J. Walker, Highly siderophile elements in Earth, Mars, the Moon, and asteroids. *Rev. Mineral Geochem.* **81**, 161–238 (2016).

6. J. van de Löcht, J. E. Hoffmann, C. Li, Z. Wang, H. Becker, M. T. Rosing, R. Kleinschrodt, C. Münker, Earth's oldest mantle peridotites show entire record of late accretion. *Geology* **46**, 199–202 (2018).

7. J. A. Coggon, A. Luguet, G. M. Nowell, P. W. U. Appel, Hadean mantle melting recorded by southwest Greenland chromitite ^{186}Os signatures. *Nat. Geosci.* **6**, 871–874 (2013).

8. T. Elliott, R. C. J. Steele, The isotope geochemistry of Ni. *Rev. Mineral. Geochemistry*. **82**, 511–542 (2017).

9. N. J. Saunders, J. Barling, J. Harvey, J. G. Fitton, A. N. Halliday, Heterogeneous nickel isotope compositions of the terrestrial mantle—Part 2: Mafic lithologies. *Geochim. Cosmochim. Acta* **317**, 349–364 (2022).

10. S.-J. Wang, W. Wang, J.-M. Zhu, Z. Wu, J. Liu, G. Han, F.-Z. Teng, S. Huang, H. Wu, Y. Wang, G. Wu, W. Li, Nickel isotopic evidence for late-stage accretion of Mercury-like differentiated planetary embryos. *Nat. Commun.* **12**, 1–7 (2021).

11. M. Klaver, D. A. Ionov, E. Takazawa, T. Elliott, The non-chondritic Ni isotope composition of Earth's mantle. *Geochim. Cosmochim. Acta* **268**, 405–421 (2020).

12. N. J. Saunders, J. Barling, J. Harvey, A. N. Halliday, Heterogeneous nickel isotopic compositions in the terrestrial mantle—Part 1: Ultramafic lithologies. *Geochim. Cosmochim. Acta* **285**, 129–149 (2020).

13. L. Gall, H. M. Williams, A. N. Halliday, A. C. Kerr, Nickel isotopic composition of the mantle. *Geochim. Cosmochim. Acta* **199**, 196–209 (2017).

14. J. Guignard, G. Quitté, M. Méheut, M. J. Toplis, F. Poitrasson, D. Connetable, M. Roskosz, Nickel isotope fractionation during metal-silicate differentiation of planetesimals: Experimental petrology and ab initio calculations. *Geochim. Cosmochim. Acta* **269**, 238–256 (2020).

15. K. Zhu, J. Barrat, A. Yamaguchi, O. Rouxel, Y. Germain, J. Langlade, F. Moynier, Nickel and chromium stable isotopic composition of ureilites: Implications for the Earth's core formation and differentiation of the ureilite parent body. *Geophys. Res. Lett.* **49**, e2021GL095557 (2022).

16. M. Willbold, T. Elliott, S. Moorbath, The tungsten isotopic composition of the Earth's mantle before the terminal bombardment. *Nature* **477**, 195–198 (2011).

17. J. B. Creech, J. A. Baker, M. R. Handler, J-P. Lorand, M. Storey, A. N. Wainwright, A. Luguet, F. Moynier, M. Bizzarro, Late accretion history of the terrestrial planets inferred from platinum stable isotopes. *Geochemical Perspect. Lett.* **3**, 94–104 (2017).

18. C. W. Dale, T. S. Kruijer, K. W. Burton, Highly siderophile element and ^{182}W evidence for a partial late veneer in the source of 3.8 Ga rocks from Isua, Greenland. *Earth Planet. Sci. Lett.* **458**, 394–404 (2017).

19. M. Fischer-Gödde, B. M. Elfers, C. Münker, K. Szilas, W. D. Maier, N. Messling, T. Morishita, M. Van Kranendonk, H. Smithies, Ruthenium isotope vestige of Earth's pre-late-veneer mantle preserved in Archaean rocks. *Nature* **579**, 240–244 (2020).

20. C. R. L. Friend, V. C. Bennett, A. P. Nutman, Abyssal peridotites >3,800 Ma from southern West Greenland: Field relationships, petrography, geochronology, whole-rock and mineral chemistry of dunite and harzburgite inclusions in the Itsaq Gneiss Complex. *Contrib. to Mineral. Petrol.* **143**, 71–92 (2002).

21. J. van de Löcht, J. E. Hoffmann, M. T. Rosing, P. Sprung, C. Münker, Preservation of Eoarchean mantle processes in ~3.8Ga peridotite enclaves in the Itsaq Gneiss Complex, southern West Greenland. *Geochim. Cosmochim. Acta* **280**, 1–25 (2020).

22. P. Waterton, J. M. Guotana, I. Nishio, T. Morishita, K. Tani, S. J. Woodland, H. Legros, D. G. Pearson, K. Szilas, No mantle residues in the Isua Supracrustal Belt. *Earth Planet. Sci. Lett.* **579**, 117348 (2022).

23. J. Zuo, A. A. G. Webb, E. J. Chin, L. Ackerman, J. Harvey, P. J. Haproff, T. Müller, Q. Wang, A. H. Hickman, D. Sorger, A. Ramírez-Salazar, Earth's earliest phaneritic ultramafic rocks: Mantle slices or crustal cumulates?, *Geophys. Geosystems.* **23**, e2022GC010519 (2022).

24. K. Szilas, P. B. Kelemen, M. T. Rosing, The petrogenesis of ultramafic rocks in the >3.7Ga Isua supracrustal belt, southern West Greenland: Geochemical evidence for two distinct magmatic cumulate trends. *Gondw. Res.* **28**, 565–580 (2015).

25. D. G. Pearson, D. Canil, S. B. Shirey, Mantle samples included in volcanic rocks: Xenoliths and diamonds. *Treatise on Geochemistry.* **2**, 171–275 (2003).

26. A. Prinzhofer, C. J. Allègre, Residual peridotites and the mechanisms of partial melting. *Earth Planet. Sci. Lett.* **74**, 251–265 (1985).

27. C. Marchesi, C. J. Garrido, M. Godard, F. Belley, E. Ferré, Migration and accumulation of ultra-depleted subduction-related melts in the Massif du Sud ophiolite (New Caledonia). *Chem. Geol.* **266**, 171–186 (2009).

28. M. F. Horan, R. J. Walker, J. W. Morgan, J. N. Grossman, A. E. Rubin, Highly siderophile elements in chondrites. *Chem. Geol.* **196**, 27–42 (2003).

29. H. Becker, M. F. Horan, R. J. Walker, S. Gao, J.-P. Lorand, R. L. Rudnick, Highly siderophile element composition of the Earth's primitive upper mantle: Constraints from new data on peridotite massifs and xenoliths. *Geochim. Cosmochim. Acta* **70**, 4528–4550 (2006).

30. T. Meisel, R. J. Walker, A. J. Irving, J. P. Lorand, Osmium isotopic compositions of mantle xenoliths: A global perspective. *Geochim. Cosmochim. Acta* **65**, 1311–1323 (2001).

31. K. Zhu, H. Becker, J. Zhu, H. Xu, Q. Man, Planetary accretion and core formation inferred from Ni isotopes in enstatite meteorites. *Geochem. Perspect. Lett.* **25**, 1–7 (2023).

32. S.-J. Wang, S.-J. Li, Y. Lin, S.-Z. Sheng, Mass-dependent nickel isotopic variations in achondrites and lunar rocks. *Geochim. Cosmochim. Acta* **350**, 16–27 (2023).

33. D. G. Pearson, N. Wittig, The formation and evolution of cratonic mantle lithosphere—Evidence from mantle xenoliths. *Treatise on Geochemistry.* **3**, 255–292 (2014).

34. Y. Xu, D. Li, D. Li, G. Dong, D. G. Pearson, J. Liu, Modification of lithospheric mantle by melts/fluids with different sulfur fugacities during the Wilson cycle: Insights from Lesvos and global ophiolitic peridotites. *J. Geophys. Res. Solid Earth*, **126**, e2021JB022445 (2021).

35. I. S. Puchtel, R. W. Nicklas, J. Slagle, M. Horan, R. J. Walker, E. G. Nisbet, M. Locmelis, Early global mantle chemical and isotope heterogeneity revealed by the komatiite-basalt record: The Western Australia connection. *Geochim. Cosmochim. Acta* **320**, 238–278 (2022).

36. S. J. Barnes, M. L. Fiorentini, Iridium, ruthenium and rhodium in komatiites: Evidence for iridium alloy saturation. *Chem. Geol.* **257**, 44–58 (2008).

37. H. Rizo, R. J. Walker, R. W. Carlson, M. Touboul, M. F. Horan, I. S. Puchtel, M. Boyet, M. T. Rosing, Early Earth differentiation investigated through ^{142}Nd, ^{182}W, and highly siderophile element abundances in samples from Isua, Greenland, *Geochim. Cosmochim. Acta.* **175**, 319–336 (2016).

38. W. D. Maier, S. J. Barnes, I. H. Campbell, M. L. Fiorentini, P. Peltonen, S. J. Barnes, R. H. Smithies, Progressive mixing of meteoritic veneer into the early Earths deep mantle. *Nature* **460**, 620–623 (2009).

39. I. S. Puchtel, J. Blichert-Toft, M. Touboul, R. J. Walker, ^{182}W and HSE constraints from 2.7 Ga komatiites on the heterogeneous nature of the Archean mantle. *Geochim. Cosmochim. Acta* **228**, 1–26 (2018).

40. I. S. Puchtel, A. Mundl-Petermeier, M. Horan, E. J. Hanski, J. Blichert-Toft, R. J. Walker, Ultra-depleted 2.05 Ga komatiites of Finnish Lapland: Products of grainy late accretion or core-mantle interaction? *Chem. Geol.* **554**, 119801 (2020).

41. I. S. Puchtel, J. Blichert-Toft, M. F. Horan, M. Touboul, R. J. Walker, The komatiite testimony to ancient mantle heterogeneity. *Chem. Geol.* **594**, 120776 (2022).

42. A. P. Nutman, C. R. L. Friend, Adjacent terranes with ca. 2715 and 2650 Ma high-pressure metamorphic assemblages in the Nuuk region of the North Atlantic Craton, southern West Greenland: Complexities of Neoarchaean collisional orogeny. *Precambrian Res.* **155**, 159–203 (2007).

43. A. P. Nutman, C. R. L. Friend, K. Horie, H. Hidaka, Chapter 3.3 The Itsaq gneiss complex of Southern West Greenland and the construction of Eoarchaean Crust at Convergent Plate Boundaries. *Dev. Precambrian Geol.* **15**, 187–218 (2007).

44. S.-Z. Sheng, S.-J. Wang, X.-M. Yang, L.-H. Chen, G. Zeng, Y. Xiao, J. Shen, X.-H. Dong, Y.-W. Lv, Sulfide dissolution on the nickel isotopic composition of basaltic rocks. *J. Geophys. Res. Solid Earth.* **127**, e2022JB024555 (2022).

45. A. Hofmann, A. Bekker, P. Dirks, B. Gueguen, D. Rumble, O. J. Rouxel, Comparing orthomagmatic and hydrothermal mineralization models for komatiite-hosted nickel deposits in Zimbabwe using multiple-sulfur, iron, and nickel isotope data. *Miner. Depos.* **49**, 75–100 (2014).

46. B. Gueguen, O. Rouxel, E. Ponzevera, A. Bekker, Y. Fouquet, Nickel isotope variations in terrestrial silicate rocks and geological reference materials measured by MC-ICP-MS. *Geostand. Geoanalytical Res.* **37**, 297–317 (2013).

47. R. S. Hiebert, A. Bekker, M. G. Houlé, O. J. Rouxel, Nickel isotope fractionation in komatiites and associated sulfides in the hart deposit, Late Archean Abitibi Greenstone Belt Canada. *Chem. Geol.* **603**, 120912 (2022).

48. L.-M. Chen, P. C. Lightfoot, J.-M. Zhu, F.-Z. Teng, Q. Duan, R. Yin, G. Wu, S.-Y. Yu, R.-Z. Hu, Nickel isotope ratios trace the process of sulfide-silicate liquid immiscibility during magmatic differentiation. *Geochim. Cosmochim. Acta* **353**, 1–12 (2023).

49. J.-P. Lorand, A. Luguet, Chalcophile and siderophile elements in mantle rocks: Trace elements controlled by trace minerals. *Rev. Mineral. Geochemistry* **81**, 441–488 (2016).

50. M. Klaver, E. S. Steenstra, M. Borchert, E. Welter, M. Wilke, J. Berndt, S. Klemme, The effect of alkalinity on Ni–O bond length in silicate glasses: Implications for Ni isotope geochemistry. *Chem. Geol.* **610**, 121070 (2022).

51. C. Lazar, E. D. Young, C. E. Manning, Experimental determination of equilibrium nickel isotope fractionation between metal and silicate from 500°C to 950°C. *Geochim. Cosmochim. Acta* **86**, 276–295 (2012).

52. A. Morbidelli, G. Libourel, H. Palme, S. A. Jacobson, D. C. Rubie, Subsolar Al/Si and Mg/Si ratios of non-carbonaceous chondrites reveal planetesimal formation during early condensation in the protoplanetary disk. *Earth Planet. Sci. Lett.* **538**, 116220 (2020).

53. P. H. Warren, Stable-isotopic anomalies and the accretionary assemblage of the Earth and Mars: A subordinate role for carbonaceous chondrites. *Earth Planet. Sci. Lett.* **311**, 93–100 (2011).

54. D. C. Rubie, V. Laurenz, S. A. Jacobson, A. Morbidelli, H. Palme, A. K. Vogel, D. J. Frost, Highly siderophile elements were stripped from Earth's mantle by iron sulfide segregation. *Science* **353**, 1141–1144 (2016).

55. M. Fischer-Gödde, T. Kleine, Ruthenium isotopic evidence for an inner Solar System origin of the late veneer. *Nature* **541**, 525–527 (2017).

56. R. C. J. Steele, T. Elliott, C. D. Coath, M. Regelous, Confirmation of mass-independent Ni isotopic variability in iron meteorites. *Geochim. Cosmochim. Acta* **75**, 7906–7925 (2011).

57. R. C. J. Steele, C. D. Coath, M. Regelous, S. Russell, T. Elliott, Neutron-poor nickel isotope anomalies in meteorites. *Astrophys. J.* **758**, 1–21 (2012).

58. M. Regelous, T. Elliott, C. D. Coath, Nickel isotope heterogeneity in the early Solar System. *Earth Planet. Sci. Lett.* **272**, 330–338 (2008).

59. M. I. Varas-Reus, S. König, A. Yierpan, J. P. Lorand, R. Schoenberg, Selenium isotopes as tracers of a late volatile contribution to Earth from the outer Solar System. *Nat. Geosci.* **12**, 779–782 (2019).

60. N. Braukmüller, F. Wombacher, C. Funk, C. Münker, Earth's volatile element depletion pattern inherited from a carbonaceous chondrite-like source. *Nat. Geosci.* **12**, 564–568 (2019).

61. Z. Wang, H. Becker, Ratios of S, Se and Te in the silicate Earth require a volatile-rich late veneer. *Nature* **499**, 328–331 (2013).

62. M. Brown, T. Johnson, Secular change in metamorphism and the onset of global plate tectonics. *Am. Mineral.* **103**, 181–196 (2018).

63. J. Tusch, C. Münker, E. Hasenstab, M. Jansen, C. S. Marien, F. Kurzweil, M. J. van Kranendonk, H. Smithies, W. Maier, D. Garbe-Schönberg, Convective isolation of hadean mantle reservoirs through archean time. *Proc. Natl. Acad. Sci. U.S.A.* **118**, e2012626118 (2020).

64. J. R. Reimink, A. Mundl-Petermeier, R. W. Carlson, S. B. Shirey, R. J. Walker, D. G. Pearson, Tungsten isotope composition of Archean crustal reservoirs and implications for terrestrial µ182W evolution. *Geochemistry, Geophys. Geosystems.* **21**, e2020GC009155 (2020).

65. R. W. Carlson, M. Garçon, J. O'Neil, J. Reimink, H. Rizo, The nature of Earth's first crust. *Chem. Geol.* **530**, 119321 (2019).

66. E. Hasenstab-Dübeler, J. Tusch, J. E. Hoffmann, M. Fischer-Gödde, K. Szilas, C. Münker, Temporal evolution of 142Nd signatures in SW Greenland from high precision MC-ICP-MS measurements. *Chem. Geol.* **614**, 121141 (2022).

67. E. Hyung, S. B. Jacobsen, The142Nd/144Nd variations in mantle-derived rocks provide constraints on the stirring rate of the mantle from the Hadean to the present. *Proc. Natl. Acad. Sci. U.S.A.* **117**, 14738–14744 (2020).

68. C. O'Neill, V. Debaille, The evolution of Hadean-Eoarchaean geodynamics. *Earth Planet. Sci. Lett.* **406**, 49–58 (2014).

69. Z. Deng, M. Schiller, M. G. Jackson, M. Millet, L. Pan, K. Nikolajsen, N. S. Saji, D. Huang, M. Bizzarro, Earth's evolving geodynamic regime recorded by titanium isotopes. *Nature* **621**, 100–104 (2023).

70. A. P. Nutman, V. R. McGregor, C. R. L. Friend, V. C. Bennett, P. D. Kinny, The Itsaq Gneiss Complex of southern West Greenland; the world's most extensive record of early crustal evolution (3900-3600 Ma). *Precambrian Res.* **78**, 1–39 (1996).

71. G. Wu, J.-M. Zhu, X. Wang, G. Han, D. Tan, S.-J. Wang, A novel purification method for high precision measurement of Ni isotopes by double spike MC-ICP-MS. *J. Anal. At. Spectrom* **34**, 1639–1651 (2019).

72. W. Li, J.-M. Zhu, D. Tan, G. Han, Z. Zhao, G. Wu, The $\delta^{60/58}$Ni values of twenty-six selected geological reference materials. *Geostand. Geoanalytical Res.* **44**, 523–535 (2020).

73. G. Wu, J.-M. Zhu, X. Wang, T. M. Johnson, Y. He, F. Huang, L.-X. Wang, S.-C. Lai, Nickel isotopic composition of the upper continental crust. *Geochim. Cosmochim. Acta* **332**, 263–284 (2022).

74. L. Zhang, W. R. Hyde, C. L. Kirkland, Y. Han, K. Szilas, Geochemical and thermodynamic constraints on Archean comagmatic volcanic and cumulate rocks from southern West Greenland. *Geochim. Cosmochim. Acta* **348**, 122–139 (2023).

75. A. Ishikawa, R. Senda, K. Suzuki, C. W. Dale, T. Meisel, Re-evaluating digestion methods for highly siderophile element and ^{187}Os isotope analysis: Evidence from geological reference materials. *Chem. Geol.* **384**, 27–46 (2014).

76. W. F. McDonough, S. -s. The composition of the Earth. *Chem. Geol.* **120**, 223–253 (1995).

77. I. S. Puchtel, M. Humayun, A. J. Campbell, R. A. Sproule, C. M. Lesher, Platinum group element geochemistry of komatiites from the Alexo and Pyke Hill areas, Ontario, Canada. *Geochim. Cosmochim. Acta.* **68**, 1361–1383 (2004).

78. I. S. Puchtel, M. Humayun, Highly siderophile element geochemistry of ^{187}Os-enriched 2.8 Ga Kostomuksha komatiites, Baltic Shield. *Geochim. Cosmochim. Acta* **69**, 1607–1618 (2005).

79. B. D. Connolly, I. S. Puchtel, R. J. Walker, R. Arevalo, P. M. Piccoli, G. Byerly, C. Robin-Popieul, N. Arndt, Highly siderophile element systematics of the 3.3Ga Weltevreden komatiites, South Africa: Implications for early Earth history. *Earth Planet. Sci. Lett.* **311**, 253–263 (2011).

80. H. Becker, C. W. Dale, Re–Pt–Os isotopic and highly siderophile element behavior in oceanic and continental mantle tectonites. *Rev. Mineral. Geochemistry.* **81**, 369–440 (2016).

81. C. Herzberg, Geodynamic information in peridotite petrology. *J. Petrol.* **45**, 2507–2530 (2004).

82. S.-s. Sun, W. F. McDonough, Chemical and isotopic systematics of oceanic basalts: Implications for mantle composition and processes. *Geol. Soc. Spec. Publ.* **42**, 313–345 (1989).

83. D. C. Rubie, D. J. Frost, U. Mann, Y. Asahara, F. Nimmo, K. Tsuno, P. Kegler, A. Holzheid, H. Palme, Heterogeneous accretion, composition and core-mantle differentiation of the Earth. *Earth Planet. Sci. Lett.* **301**, 31–42 (2011).

84. M. I. Smoliar, R. J. Walker, J. W. Morgan, Re-Os ages of group IIA, IIIA, IVA, and IVB iron meteorites. *Science* **271**, 1099–1102 (s).

85. R. J. Walker, S. B. Shirey, O. Stecher, Comparative Re–Os, Sm–Nd and Rb–Sr isotope and trace element systematics for Archean komatiite flows from Munro Township, Abitibi Belt, Ontario, *Earth Planet. Sci. Lett.* **87**, 1–12 (1988).

86. S. J. Wang, R. L. Rudnick, R. M. Gaschnig, H. Wang, L. E. Wasylenki, Methanogenesis sustained by sulfide weathering during the Great Oxidation Event. *Nat. Geosci.* **12**, 296–300 (2019).

87. F.-Y. Wu, R. J. Walker, Y.-H. Yang, H.-L. Yuan, J.-H. Yang, The chemical-temporal evolution of lithospheric mantle underlying the North China Craton. *Geochim. Cosmochim. Acta* **70**, 5013–5034 (2006).

88. J. Liu, R. L. Rudnick, R. J. Walker, S. Gao, F.-Y. Wu, P. M. Piccoli, H. Yuan, W. Xu, Y.-G. Xu, Mapping lithospheric boundaries using Os isotopes of mantle xenoliths: An example from the North China Craton. *Geochim. Cosmochim. Acta* **75**, 3881–3902 (2011).

89. L. Reisberg, J.-P. Lorand, Longevity of sub-continental mantle lithosphere from osmium isotope systematics in orogenic peridotite massifs. *Nature* **376**, 159–162 (1995).

90. A. E. Saal, E. Takazawa, F. A. Frey, N. Shimizu, S. R. Hart, Re–Os isotopes in the Horoman peridotite: Evidence for refertilization? *J. Petrol.* **42**, 25–37 (2001).

91. D. G. Pearson, G. J. Irvine, D. A. Ionov, F. R. Boyd, G. E. Dreibus, Re–Os isotope systematics and platinum group element fractionation during mantle melt extraction: A study of massif and xenolith peridotite suites. *Chem. Geol.* **208**, 29–59 (2004).

92. J. E. Snow, G. Schmidt, Proterozoic melting in the northern peridotite Massif, Zabargad Island: Os isotopic evidence. *Terra Nov.* **11**, 45–50 (1999).

93. Z. Wang, H. Becker, T. Gawronski, Partial re-equilibration of highly siderophile elements and the chalcogens in the mantle: A case study on the Baldissero and Balmuccia peridotite massifs (Ivrea Zone, Italian Alps). *Geochim. Cosmochim. Acta* **108**, 21–44 (2013).
